# A collagen amino acid composition supplementation reduces biological age in humans and increases health and lifespan in vivo

**DOI:** 10.1038/s41514-025-00280-7

**Published:** 2025-11-20

**Authors:** Alexander Dakhovnik, Matilde Mantovani, Marie Knufinke, Victoria Brügger, Intan Pramono, Cyril Statzer, Babak Saravi, Oleksandr Demidenko, Yelena Budovskaya, Claudia Bettina Rümmelein, Sophie Chabloz, Collin Y. Ewald

**Affiliations:** 1 Laboratory of Extracellular Matrix Regeneration, Institute of Translational Medicine, Department of Health Sciences and Technology, ETH Zürich, Schwerzenbach, Switzerland; 2 Avea Life AG, Bahnhofplatz, Zug, Switzerland; 3 Hautwerk AG, Zurich, Switzerland; 4 Department of Dermatology, University Hospital Zurich, Zurich, Switzerland; 5 Department of Oral, Maxillofacial and Facial Plastic Surgery, Medical Faculty and University Hospital Düsseldorf, Heinrich-Heine-University Düsseldorf, Düsseldorf, Germany; 6 TruMe Inc., Alameda, CA USA

**Keywords:** Biomarkers, Cell signalling, Epigenetics, Translational research, Ageing

## Abstract

Collagen supplementation has gained attention with increasing claims regarding its beneficial effects on healthy aging based on clinical observations and lifespan extension in pre-clinical models; however, how and which part of an ingested collagen promotes healthy longevity is unknown. Here, we identified the minimal required unit of ingested collagen, which consists of the proper ratio of three glycine to one proline to one hydroxyproline that was sufficient to increase the motility-healthspan and lifespan of *C. elegans*, as well as collagen homeostasis in human fibroblasts in vitro. Supplementation in 20-month-old mice improved grip strength and prevented age-related fat accumulation. In a clinical observational trial (ISRCTN93189645, 03.07.2025), oral supplementation in humans demonstrated improved skin features within three months and a reduction in biological age by 1.4 years (*p* = 0.04) within 6 months. Thus, a ratio of three amino acids elicits evolutionarily conserved health benefits from ingested collagens.

## Introduction

Collagen is one of the most abundant proteins, making up 12–17% of the total proteins in mammals^1,2^. Therefore, collagen is a major component when consuming meat. Oral intake of collagen is digested by gastric enzymes, and the resulting collagen-derived di- and tripeptides are transported through the transmembrane peptide transporter (PEPT-1) into enterocytes, where these peptides are further broken down to amino acids and then released into the bloodstream, yielding about 39–60% absorption of the collagen amnio acids depending on how much the collagen was crosslinked^3,4^.

Over the past few years, collagen supplementation has gained popularity for its potential health benefits. In 2024, the global collagen market size was estimated at approximately $ 9.4 billion, with a compound annual growth rate (CAGR) of 5.4%. It is projected to be roughly 16 billion USD by 2033^5^, suggesting an increasing consumer demand. However, the underlying science of the mechanism of action for health benefits upon collagen supplementation is lacking.

As we age, collagen production decreases by approximately 1–1.5% per year in human skin^6,7^. This decline is a significant factor in the development of fine lines and deeper wrinkles. Additional signs of skin aging include reduced elasticity, reduced hydration, and increased transepidermal water loss^8^. Not surprisingly, assuming that collagens are the building block for the skin, oral collagen peptide supplementation improved skin hydration and elasticity and even reduced the appearance of wrinkles and cellulitis within 12 weeks in four independent randomized, double-blinded, placebo-controlled clinical trials^9–12^. Similarly, a significant constituent of nails, tendons, and bones is collagen, and oral collagen peptide intake for 12–14 weeks improved brittle nails and nail growth, increased patellar tendon cross-sectional area, bone mineral density, and bone collagen synthesis^13–15^. Even in young, active men and women suffering from knee joint discomfort, oral collagen peptide supplementation reduced activity-related joint pain^16^. Furthermore, mechanical wear and tear on joints during aging leads to osteoarthritis or age-related rheumatoid arthritis, where the body’s immune system attacks its joints. Oral collagen intake for 4–6 months decreases joint pain and improves physical activity^17–19^. In line with physical activity improvements, muscle mass declines during aging, leading to sarcopenia, and it is well established that resistance training counteracts sarcopenia^20^, but resistance training combined with collagen peptide supplementation showed further improvements in increasing lean body mass (i.e., muscles) in young and old sarcopenic men^21–25^, decreased fat mass and increased lean mass and hand-grip strength in pre-menopausal women in double-blinded randomized clinical trials^15^. Even without exercise, collagen peptide intake increased glycine, proline, and hydroxyproline levels in blood plasma and increased muscle connective protein synthesis rates in young men in a randomized, double-blinded, parallel-designed clinical study^26^. Lastly, type 2 diabetic patients with and without hypertension who took collagen peptides showed significant reduction in fasting blood glucose levels, hemoglobin A1c (HbA1c), diastolic blood pressure, total cholesterol, low-density lipoprotein, and triglyceride levels, and improved insulin sensitivity index in randomized, double-blinded clinical studies, suggesting improvements in glucose and lipid metabolism^27–29^.

In preclinical models and laboratory model organisms, collagen supplementation increases the health and lifespan of rats^30^, *Drosophila*^31^, and *C. elegans*^32,33^, suggesting that collagen supplementation might be a “longevity pill”. There are at least 28 types of collagens from 44 collagen genes in humans and mice^34^ and five types of collagen from 184 collagen genes in *C. elegans*^35^. Different collagen types vary in molecular assembly, cellular components, and physiological distribution, each fulfilling specialized roles in the body^34^. Furthermore, cleavage products of collagens^36^ (matrikines) can act as signaling molecules to prevent or promote diseases, such as cancer^34^. Thus, begging the question of which part of collagen is sufficient to induce these healthspan and longevity effects, mechanistically.

In this study, we identify that simply the major amino acids of collagen in its proper ratio of 3 glycine : 1 proline : 1 hydroxyproline are sufficient to increase the lifespan of *C. elegans* and collagen homeostasis of *C. elegans* and human skin cells in vitro. Supplementing these collagen amino acids is safe in geriatric mice (20–26 months of age), improving grip strength and preventing age-related fat accumulation. In our observational trial, we demonstrate that combining these collagen amino acids is safe in humans, improves skin appearance, and reduces biological age by 1.4 years (*p* = 0.04) upon 6 months of oral supplementation.

## Results

### A combination of three collagen amino acids prolonged collagen homeostasis during *C. elegans* aging

**Fig. 1:**
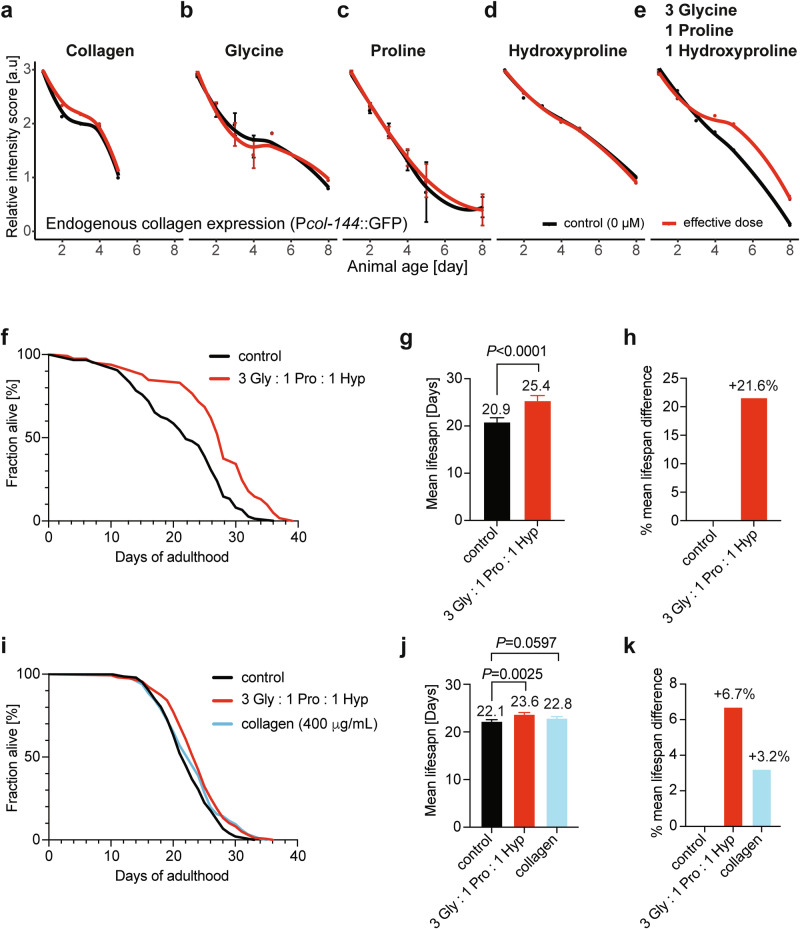
Supplementation of 3 Gly : 1 Pro : 1 Hyp prolonged endogenous collagens expression during aging and promoted longevity. Synchronized L4 transgenic *C. elegans* driving GFP with the collagen *col-144* promoter (LSD2002 P*col-144*::GFP) were placed on culturing plates containing heat-killed OP50 bacteria food with either rat tail collagen (**a**), glycine (**b**), proline (**c**), hydroxyproline (**d**), or a mixture of [3 Gly : 1 Pro : 1 Hyp] (**e**). GFP intensity was scored during aging at 25 °C. *N* > 100 per condition. See Supplementary Fig. 1a–e and Supplementary Table 1 for additional doses, the definition of effective dose (*), and raw data. **f**–**h** Supplementation of [3 Gly : 1 Pro : 1 Hyp] starting from young adulthood increased the lifespan of LSD2002 P*col-144*::GFP *C. elegans* (**f**) and also prolonged endogenous collagen expression (Supplementary Fig. 1g). **f** Survival curves. *N* > 100 per condition. **g** Graph of mean ± SEM lifespan. **h** Graph of percent mean lifespan increase. **i**–**k** Supplementation of rat tail collagen or [3 Gly : 1 Pro : 1 Hyp] starting from young adulthood increased the lifespan of temperature-sensitive wild-type background TJ1060 *C. elegans*. **i** Survival curves. *N* > 100 per condition. **j** Graph of mean ± SEM lifespan. **k** Graph of percent mean lifespan increase. For **f**–**k**, statistical analysis, replicates, and raw data are in Supplementary Table 2.

Collagen supplementation is sufficient to increase the lifespan of *C. elegans* and rats^33,37^. However, which part of the collagen promotes longevity is unknown. To determine the beneficial components of collagens, we used a recently established surrogate biomarker for longevity^38^. Similar to humans, collagen expression progressively declines during *C. elegans* aging^39^. We used a transcriptional reporter expressing green fluorescent protein (GFP) driven by the collagen *col-144* promoter (P*col-144*::GFP)^38^, which expression progressively declined within 6–8 days of adulthood (Fig. 1a–e). We observed that supplementing *C. elegans* with rat tail collagen slightly increased endogenous *col-144* expression during days 2–4 of adulthood (Fig. 1a, Supplementary Fig. 1a and Supplementary Table 1). The major components of collagens are their characteristic triple helices repeats of [Glycine-X-Y]_n_, whereby X is usually proline, and Y is hydroxyproline^34,40^. Therefore, as a control experiment, we fed *C. elegans* with various doses of either glycine, proline, or hydroxyproline, which did not alter *col-144* expression during aging (Fig. 1b–d, Supplementary Fig. 1b–d and Supplementary Table 1). However, glycine is the smallest amino acid, with hydrogen as its side chain. Glycine must occur at every third position in the collagen repeats to form a proper collagen triple helix^34,40^. Therefore, a common feature of collagen composition is that glycine content is three times higher than that of other amino acids^34,40^. Surprisingly, when we adjusted the feeding ratio 3 to 1 to 1 of glycine, proline, and hydroxyproline [3 Gly : 1 Pro : 1 Hyp], we found a stark prolongation of *col-144* expression during *C. elegans* aging (Fig. 1e, Supplementary Fig. 1e–h and Supplementary Table 1).

### A composition ratio of 3 Gly : 1 Pro : 1 Hyp is superior in increasing lifespan compared to collagen supplementation

To determine whether the prolonged collagen homeostasis of [3 Gly : 1 Pro : 1 Hyp] supplementation corresponds to lifespan benefits, we measured the lifespan of *C. elegans* fed this ratio of amino acids post-development, starting from young adulthood. We found that [3 Gly : 1 Pro : 1 Hyp] supplementation significantly increased lifespan by 6–27% across five independent trials, even on heat-inactivated bacteria (Fig. 1f–k, Supplementary Fig. 1i–k and Supplementary Table 2). In contrast to previous studies where 5–1000 µM glycine but not 5–10 mM glycine extended lifespan or 1–10 mM proline extended lifespan^41,42^, in our experimental setup supplementing individually 5 mM glycine or 5 mM proline, or 5 mM hydroxyproline were insufficient to increase lifespan (Supplementary Fig. 1l–n and Supplementary Table 2). To compare this increase in lifespan with collagen supplementation, we fed *C. elegans* with 400 µg/ml of rat tail collagen supplemented in their food and assayed both conditions, whole collagen versus [3 Gly : 1 Pro : 1 Hyp] supplementation, in parallel. We found that collagen supplementation increased mildly the mean lifespan but robustly the maximum lifespan, whereas [3 Gly : 1 Pro : 1 Hyp] supplementation increased both the mean and maximum lifespan (Fig. 1i–k and Supplementary Table 2). Thus, the [3 Gly : 1 Pro : 1 Hyp] supplementation outperformed collagen supplementation in promoting longevity.

### A broad range of supplementing with different Gly : Pro : Hyp ratios promotes physical performance and longevity

**Fig. 2:**
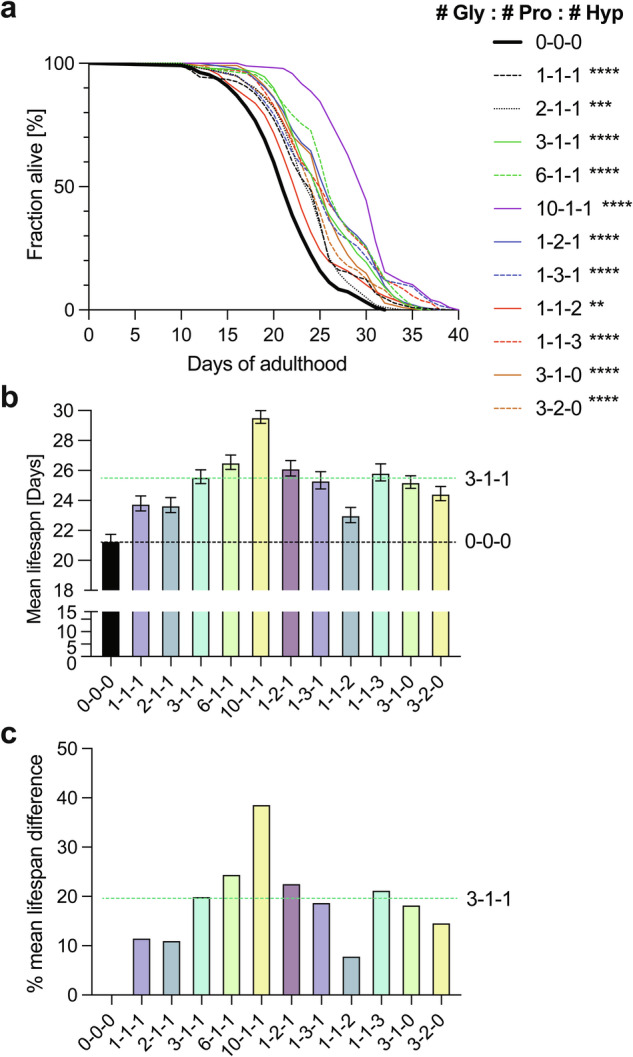
Different ratios sufficient to increase lifespan. **a**–**c** Synchronized day-2 adult *C. elegans* were placed on culturing plates containing heat-killed OP50 bacteria food with different ratios of [Gly : Pro : Hyp]. **a** Survival curve. **b** Mean ± SEM, **c** percent mean lifespan change relative to no supplementation of [Gly : Pro : Hyp] (i.e., 0-0-0). *N* > 100 per condition. For details, statistics, and raw data, see Supplementary Table 2.

Next, we asked what the optimal combination and ratio for this healthspan and lifespan increase is. By increasing glycine content (# Gly : 1 Pro : 1 Hyp, whereby # = 1, 2, 3, 6, 10), we found a dose-dependent increase in lifespan (Fig. 2a and Supplementary Table 2). Similarly, increasing proline levels [1 Gly : 2 Pro : 1 Hyp] or [1 Gly : 3 Pro : 1 Hyp] showed increased lifespan compared to [1 Gly : 1 Pro : 1 Hyp] and was comparable to increased glycine of [3 Gly : 1 Pro : 1 Hyp] or [6 Gly : 1 Pro : 1 Hyp] (Fig. 2a and Supplementary Table 2). Furthermore, increasing only hydroxyproline [1 Gly : 1 Pro : 3 Hyp] showed an increased lifespan compared to [1 Gly : 1 Pro : 1 Hyp] and was comparable to [3 Gly : 1 Pro : 1 Hyp] (Fig. 2a and Supplementary Table 2). However, omitting hydroxyproline [3 Gly : 1 Pro : 0 Hyp] or [3 Gly : 2 Pro : 0 Hyp] showed less lifespan increase compared [3 Gly : 1 Pro : 1 Hyp] (Fig. 2a and Supplementary Table 2), suggesting that a combination of all three amino acids has synergistic benefits. Furthermore, combinations of [3 Gly : 1 Pro : 1 Hyp], [1 Gly : 3 Pro : 1 Hyp], or [1 Gly : 1 Pro : 3 Hyp] showed similar longevity, which the individual contribution of each amino acid can not explain and suggests that a 3 to 1 to 1 ratio for any of these amino acids might elicit this beneficial response.

The lifespans shown above were performed on solid media, where *C. elegans* are typically less motivated to move and rest in the food during aging^43^. To measure their active movement capability during aging (i.e., healthspan), we assayed their thrashing rates in liquids (i.e., swimming) during their entire lifespan. We found that most ratios exhibited prolonged movement capability during aging in at least one of the two trials (Supplementary Fig. 2a, b and Supplementary Table 3). The [3 Gly : 1 Pro : 1 Hyp] consistently improved activity during aging. In one trial, the only two combinations showing higher swimming rates during mid-life than [3 Gly : 1 Pro : 1 Hyp] were [3 Gly : 1 Pro : 0 Hyp] and [1 Gly : 1 Pro : 3 Hyp] (Supplementary Fig. 2b and Supplementary Table 3). Interestingly, at the very old age of >24 days of adulthood at the end of the lifespan, [3 Gly : 1 Pro : 1 Hyp] fed *C. elegans* were the most active (Supplementary Fig. 2 and Supplementary Table 3).

Taken together, this suggests that increasing the levels of each amino acid is sufficient to increase lifespan further, but combining those three amino acids in a 3 to 1 to 1 ratio has synergistic effects that are greater than the sum of their parts.

### Synergistic longevity by combining α-ketoglutarate and 3 Gly : 1 Pro : 1 Hyp

Hydroxyproline is essential for collagen triple helix stability^40^. During collagen synthesis in the endoplasmic reticulum, the prolyl-hydroxylase adds hydroxy to the proline in the collagen chain^40^. A key and rate-limiting co-factor for prolyl-hydroxylase activity is the Krebs cycle intermediate α-ketoglutarate, which promotes collagen stability^44,45^. Supplementing human dermal fibroblasts with α-ketoglutarate increases collagen production^45^. Supplementing *C. elegans* or mice with α-ketoglutarate increases their lifespan^46,47^. We confirmed that supplementing *C. elegans* with α-ketoglutarate from young adulthood increased their lifespan (Supplementary Fig. 3a–c and Supplementary Table 2). Intriguingly, combining α-ketoglutarate with [3 Gly : 1 Pro : 1 Hyp] showed additive effects on their longevity (Supplementary Fig. 3a and Supplementary Table 2). We wondered whether the longevity upon α-ketoglutarate supplementation would be mediated through its co-factor activity for prolyl-hydroxylase. We used a mutant *dpy-18(ok162)* of the prolyl-4-hydroxylase alpha subunit that eliminates its propyl-4-hydroxylase activity^44^. The longevity upon α-ketoglutarate supplementation is completely abolished in *dpy-18(ok162)* mutants, suggesting that prolyl-hydroxylase activity is required for α-ketoglutarate-induced longevity (Supplementary Fig. 3b and Supplementary Table 2). Intriguingly, [3 Gly : 1 Pro : 1 Hyp] supplementation was still sufficient to increase lifespan (Supplementary Fig. 3b and Supplementary Table 2). These results suggest that α-ketoglutarate and [3 Gly : 1 Pro : 1 Hyp] work through parallel pathways for their additive and synergistic effects on promoting healthy aging.

### Conserved extracellular matrix enhancement of [3 Gly : 1 Pro : 1 Hyp] in human skin cells

**Fig. 3:**
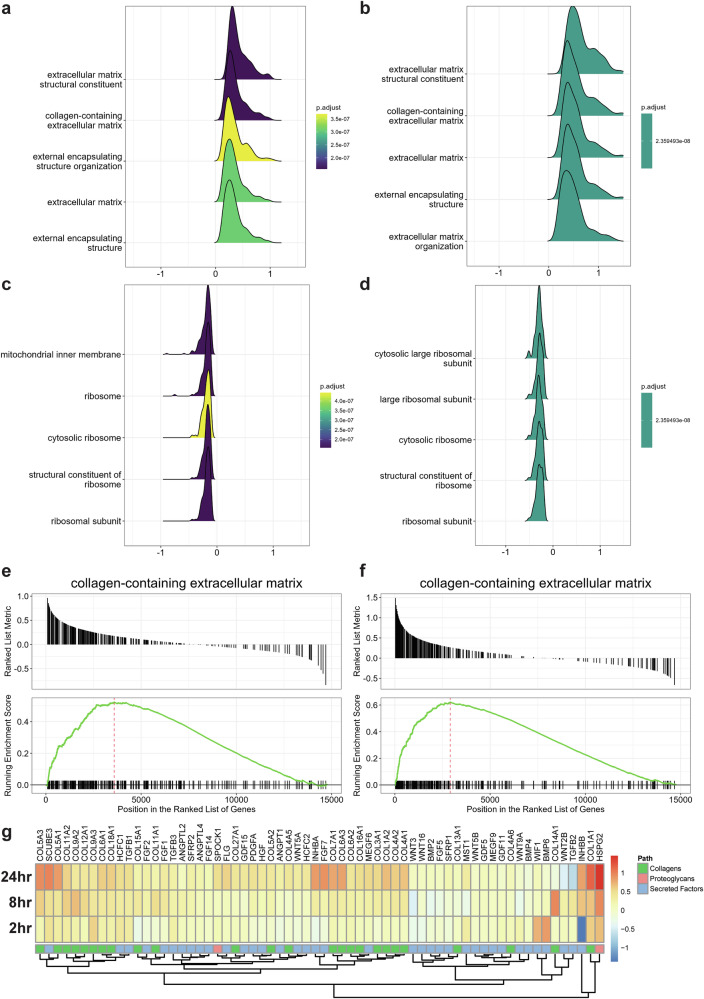
Treatment of primary human dermal fibroblasts with 3 Gly : 1 Pro : 1 Hyp increased collagen-related gene expression. Gene set enrichment analysis results show the top 5 GO pathways with positive enrichment scores at 8 h (**a**) and 24 h of treatment (**b**). The *X*-axis represents log2 fold change. Gene set enrichment analysis results show the top 5 GO pathways with negative enrichment scores at 8 h (**c**) and 24 h of treatment (**d**). The *X*-axis represents log2 fold change. Gene set enrichment plot for the GO collagen-containing extracellular matrix pathway at 8 h (**e**) and 24 h of treatment (**f**). **g** Heatmap showing log2 fold change vs. respective timepoint-matched controls of matrisome genes annotated as collagens, proteoglycans, or secreted factors.

Supplementing [3 Gly : 1 Pro : 1 Hyp] prolonged collagen expression during aging and increased the lifespan of *C. elegans*. Oral supplementation of collagen peptides increased collagen type I mRNA and protein levels in the dorsal skin of aged mice^48^. Similarly, oral administration of radioactive-labeled gelatin hydrolysate was incorporated into various tissues and found to be twice as high in cartilage compared to control in mice^49^, suggesting that orally-provided collagen components might be deposited and reutilized in collagen-rich connective tissue. Furthermore, in a human double-blind, placebo-controlled study, 8 weeks of oral intake of collagen peptides increased type I collagen levels in the skin^50^. Directly supplementing osteoblastic cells or chondrocytes with collagen peptides increases collagen mRNA levels or type II collagen protein levels in vitro, respectively^51^. Moreover, supplementing with human collagen COL7A1 peptides increases diverse collagen mRNA in primary human dermal fibroblasts and collagen COL1A1 protein secretion of HaCaT keratinocytes^37^. Hence, we hypothesized that it is likely that supplementing 3 Gly : 1 Pro : 1 Hyp] to fibroblasts would also increase endogenous collagen mRNA production. To gain mechanistic insights into how [3 Gly : 1 Pro : 1 Hyp] supplementation affects human cells in vitro, we performed RNA sequencing upon 2 h, 8 h, and 24 h of incubation of human dermal fibroblasts by supplementing with 3 mM glycine, 1 mM proline, and 1 mM hydroxyproline. Gene set enrichment analysis (GSEA) showed that at 8 h and 24 h, the most significantly enriched gene sets with positive enrichment scores were involved in collagen and extracellular matrix homeostasis, including “extracellular matrix structural constituent” and “collagen-containing extracellular matrix” (Fig. 3a, b and Supplementary Table 4). The most significantly enriched gene sets with negative enrichment scores primarily involved mitochondrial and ribosomal processes and included “mitochondrial inner membrane” and “ribosomal subunit” (Fig. 3c, d). The overall predominant signature from the GSEA was an upregulation in collagen and extracellular matrix-associated processes, a prominent downregulation of ribosomal components, and a less robust downregulation of mitochondrial-associated pathways (Supplementary Fig. 4a, b). The upregulation of collagen-related processes was the most robust signature in the dataset, with a highly consistent increase in the expression of most extracellular matrix-associated genes at the 8 and 24-h time points (Fig. 3e, f). This trend was also evident when the ‘matrisome’, or set of genes associated with extracellular matrix structure and remodeling processes, was examined. Overall upregulation was observed across all matrisome domains but was particularly pronounced in collagens (Fig. 3g). Upregulation was also seen in glycoproteins and genes involved in extracellular matrix remodeling processes (Supplementary Fig. 4c). In line with the observed prolonged collagen homeostasis in aged *C. elegans* and with previous collagen peptide supplementation on human cells, this confirms that [3 Gly : 1 Pro : 1 Hyp] supplementation promotes ECM homeostasis of human cells in vitro.

### 3 Gly : 1 Pro : 1 Hyp supplementation is safe in old male mice

**Fig. 4:**
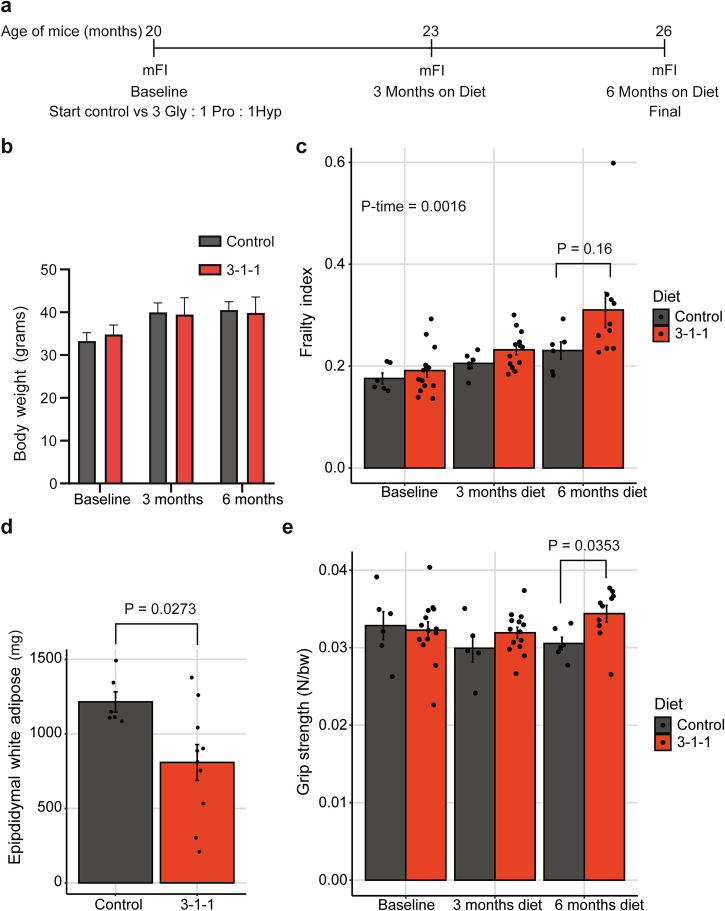
3 Gly : 1 Pro : 1 Hyp improved grip strength and reduced white adipose tissue in old mice. **a** Schematic representation of the experimental timeline starting with 20-month-old mice. **b** Body weights from baseline to 6 months for male wild-type C57BL/6J showed no difference between control-fed and [3 Gly : 1 Pro : 1 Hyp] supplementation. **c** Frailty index from baseline to 6 months for male wild-type C57BL/6J showed no difference between control-fed and [3 Gly : 1 Pro : 1 Hyp] supplementation. **d** Epididymal white adipose tissue mass was reduced after 6 months of [3 Gly : 1 Pro : 1 Hyp] supplementation. **e** Grip strength was increased at 6 months of [3 Gly : 1 Pro : 1 Hyp] supplementation. **b**–**e** Data points represent individual mice. For raw data and additional statistics, see Data Source File 1.

Next, we wanted to know whether supplementing [3 Gly : 1 Pro : 1 Hyp] is safe in old mice. We conducted a study with *n* = 20 20-month-old C57BL/6 male mice and followed them for 6 months (Fig. 4a). The mouse experiments did not include isonitrogenous or amino acid‑matched controls, which presents a potential concern for amino acid imbalance. Nevertheless, a physiologic effect of amino acid imbalance generally requires greater changes in amino acid intake than in our study, with the [3 Gly : 1 Pro : 1 Hyp] formulation at modest doses, which altered total protein intake by only ~5% compared to a standard diet. We found that the [3 Gly : 1 Pro : 1 Hyp] supplementation was well tolerated as body weights, organ weights, and food intake remained similar among groups, with no indication of amino acid imbalance response (Fig. 4b, Data Source File 1). We monitored mouse frailty from the baseline at 20 months of age to 26 months (Fig. 4c). Control-fed and [3 Gly : 1 Pro : 1 Hyp] mice exhibited a significant increase in the frailty index over these 6 months with no significant differences between groups (Fig. 4c). During aging, mice accumulated visceral fat^52^, which was significantly lower in the [3 Gly : 1 Pro : 1 Hyp] group after 6 months (Fig. 4d). Furthermore, mice, like humans, have reduced muscle function during aging^53^. However, [3 Gly : 1 Pro : 1 Hyp] supplementation maintained grip strength over these 6 months (Fig. 4e). These results suggest that [3 Gly : 1 Pro : 1 Hyp] supplementation at a dose of 100 ppm in food is safe for old mice, and further studies need to be conducted to determine the effects of [3 Gly : 1 Pro : 1 Hyp] supplementation on healthspan and lifespan in preclinical rodent models.

### 3 Gly : 1 Pro : 1 Hyp in combination with additional supplements as a new formulation to go into humans

Given the positive results of [3 Gly : 1 Pro : 1 Hyp] supplementation for extending *C. elegans* health and lifespan, promoting collagen homeostasis also in human skin cells, and [3 Gly : 1 Pro : 1 Hyp] supplementation being safe and well tolerated in old mice, we next worked on a formulation to maximize potential health benefits in humans. To support collagen synthesis, vitamin C is essential^40^, and with acerola fruit juice extract, 48 mg of vitamin C is provided per serving. In a randomized clinical trial, the antioxidant astaxanthin, in combination with collagen peptides, improved facial skin elasticity and hydration and reduced matrix metalloproteinase expression while increasing endogenous collagen expression^54^, suggesting enhanced collagen homeostasis. Furthermore, supplementing astaxanthin in yeast^55^, *C. elegans*^56,57^, *Drosophila*^58^, and male mice^59^ increased their lifespan^60^. We provide 4 mg of astaxanthin per serving in the form of algae powder. Lastly, given our additive lifespan effects with the combination of alpha-ketoglutarate and [3 Gly : 1 Pro : 1 Hyp] supplementation for extending *C. elegans’* lifespan, we provide 1 g of calcium alpha-ketoglutarate per serving. With these additional supporting ingredients, we generated a novel formulation, which we will refer to throughout the text as a “collagen activator”, to test its safety and efficacy in humans.

### Safety assessment and descriptive statistics of participants’ characteristics

**Fig. 5:**
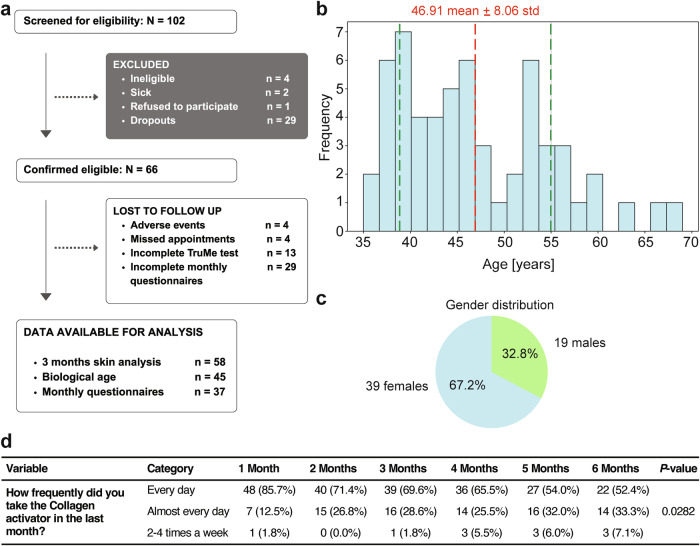
Clinical trial flowchart and age and sex distribution. **a** Scheme illustrating the flow of the participants through the observational study. **b** Age distribution of participants. **c** Gender distribution of participants. **d** Demographic and baseline characteristics, with descriptive statistics on dietary habits, supplement use, lifestyle, and health-related behaviors. Additional data, analysis, and statistics are provided in Data Source File 1.

We screened 102 participants and confirmed that 66 participants were eligible for the study (Fig. 5a). All eligible participants were included in the intervention group and received the Collagen Activator. The dropout rate due to adverse events was 6%, with four out of 66 participants experiencing mild, transient side effects that resolved without intervention (Fig. 5a). Specifically, three participants reported rosacea, characterized by facial redness and mild skin rash, while one experienced adverse intestinal reactions. All four participants who dropped out experienced these adverse effects after 2–3 months of Collagen Activator intake, suggesting a low likelihood of an acute effect of Collagen Activator towards these events and, in general, that the Collagen Activator was safe and well-tolerated. Of the 66 participants confirmed as eligible, 37 completed the monthly questionnaires. After 3 months, 58 participants underwent the three skin analyses, and 45 completed the biological age test at 6 months (Fig. 5a). The 58 participants were spread across a range of 35–68 years of age. The mean age of participants was 46.91 years ± 8.06 (95% CI: 44.80–46.48) (Fig. 5b, Data Source File 1). The cohort consisted of 39 females (67.2%) and 19 males (32.8%) (Fig. 5c). The demographic and baseline characteristics of the participants were assessed using the questionnaire. The mean body mass index (BMI) was 22.28 kg/m^2^ (21.43 for females and 23.99 for males), with a standard deviation of 2.79 kg/m^2^, indicating a relatively homogeneous distribution of BMI values across the cohort (Table 1). The majority of participants (88%) were White/Caucasians (Table 1). Furthermore, 83% of participants hold a university degree, 69% never smoked, and only 4 participants occasionally smoked, the rest all quit smoking, one-third did not drink any alcohol, and two-thirds reported either 1–2 or 3–4 drinks per week, 66% reported taking supplements, almost half of the participants exercise 3–4 times a week with 88% moderate to lightly intensity and only 2 participants reported to be sedentary (Table 1 and Data Source File 1). Regarding the adherence to Collagen Activator consumption, a total of 86% of 56 participants reported daily Collagen activator intake in the first month, which decreased to 52% at month 6 of the remaining 39 participants who filled in the questionnaire (*p* = 0.028, Fig. 5d). Taken together, our cohort, with an average age in the mid-forties, is considered young, given that this is when age-related phenotypes begin to appear in humans. Furthermore, our cohort is exceptionally well-educated and has healthy lifestyles.

**Table 1:** Demographic and baseline characteristics

Variable	Category	Value
BMI		22.28 ± 2.79
Ethnicity	White	87.9% (*n* = 51)
	Hispanic or Latino	5.2% (*n* = 3)
	Asian	3.4% (*n* = 2)
	Black or African American	1.7% (*n* = 1)
	Not specified	1.7% (*n* = 1)
Education	University Degree	82.8% (*n* = 48)
	Vocational Training	12.1% (*n* = 7)
	Secondary School or Less	3.4% (*n* = 2)
	Not specified	1.7% (*n* = 1)
Dietary pattern	Flexitarian	31.0% (*n* = 18)
	Omnivore	41.4% (*n* = 24)
	Other	15.5% (*n* = 9)
	Pescatarian	5.2% (*n* = 3)
	Vegan	1.7% (*n* = 1)
	Vegetarian	3.4% (*n* = 2)
Supplement use	No	32.8% (*n* = 19)
	Yes	65.5% (*n* = 38)
Alcohol consumption	1–2 drinks	31.0% (*n* = 18)
	3–4 drinks	31.0% (*n* = 18)
	5–8 drinks	6.9% (*n* = 4)
	None	29.3% (*n* = 17)
Smoking status	No, never	69.0% (*n* = 40)
	No, I quit	22.4% (*n* = 13)
	Yes, occasionally	6.9% (*n* = 4)
Exercise frequency	1–2 times a week	36.2% (*n* = 21)
	3–4 times a week	44.8% (*n* = 26)
	5–6 times a week	8.6% (*n* = 5)
	Daily	1.7% (*n* = 1)
	Less than once a week	6.9% (*n* = 4)
Physical activity level	Extremely active	1.7% (*n* = 1)
	Lightly active	37.9% (*n* = 22)
	Moderately active	50.0% (*n* = 29)
	Sedentary	3.4% (*n* = 2)
	Very active	5.2% (*n* = 3)

### Collagen Activator improves skin texture, hydration, and elasticity

**Fig. 6:**
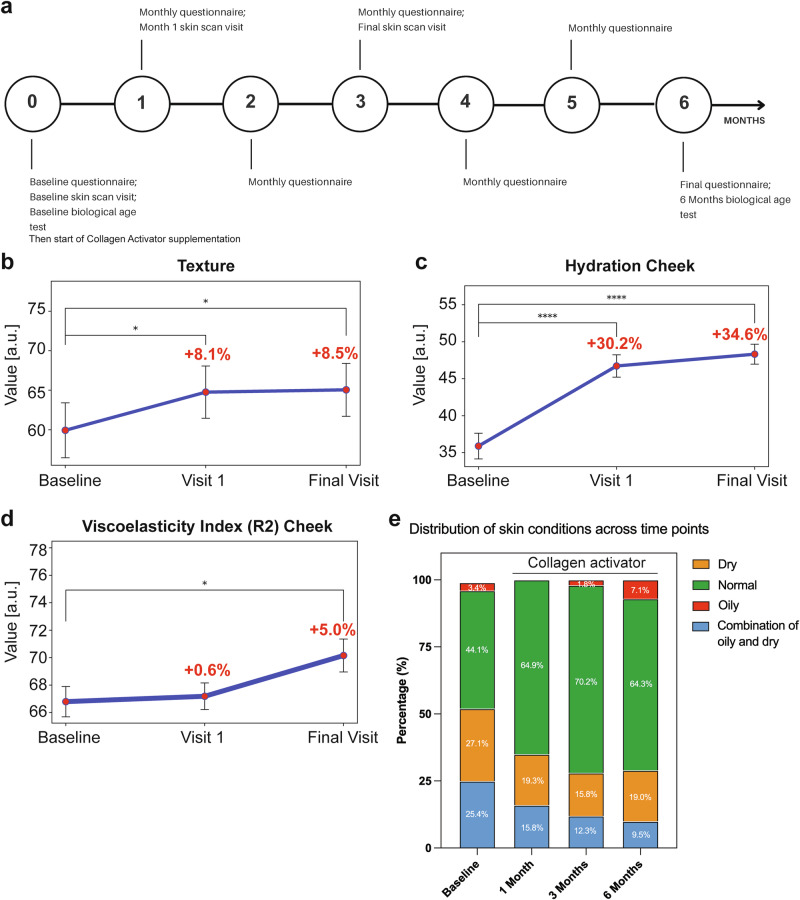
Changes in skin parameters over time upon Collagen Activator intake. **a** Study timeline. **b** Changes in texture from baseline through the final visit. Each subplot represents the respective parameter’s mean and 95% CI at each time point. Significant differences between time points are indicated by asterisks (*p < 0.05, **p < 0.01, ***p < 0.001, ****p < 0.0001). **c** Changes in cheek hydration from baseline through to the final visit. Hydration levels are expressed in relative corneometer units (AU), with data shown as mean and 95% CI. Significant differences between time points are indicated by asterisks (*p < 0.05, **p < 0.01, ***p < 0.001, ****p < 0.0001). **d** Changes in skin elasticity (R2) over time. This figure displays the R2 viscoelasticity index measured on the cheek at baseline, visit 1, and final visit. Data are presented as mean and 95% CI. Significant differences between time points are indicated by asterisks (*p < 0.05, **p < 0.01, ***p < 0.001, ****p < 0.0001). **e** Distribution of self-reported skin conditions over time. Skin conditions are categorized into four categories: “Combination of oily and dry,” “Dry,” “Normal (neither oily nor dry),” and “Oily”. Additional data, analysis, and statistics are provided in Data Source File 1.

At time point 0 (baseline), participants completed a questionnaire, a skin scan of the cheek, forearm, and upper arm via Visia scan, Cutometer and Corneometer analysis at Hautwerk Clinic in Zurich, and provided saliva for an epigenetic age test^61^ (Fig. 6a). Participants filled out the questionnaire every month, and during months 1 and 3, they visited Hautwerk Zurich for skin analysis. Participants completed the questionnaire at month 6 and provided a saliva sample to estimate their biological age (Fig. 6a).

Not surprisingly, for the short duration of the 3-month study, wrinkle severity showed a downward trend from baseline (34.07%, 95% CI: 26.80–41.34%) to the final visit (30.00%, 95% CI: 23.80–36.20%, Data Source File 1). However, this change did not reach statistical significance (*p* = 0.2640; Data Source File 1). By contrast, we found that supplementing Collagen Activator improved skin texture after 1 month and improved it for the rest of the study. The skin texture score increased significantly from baseline (59.93%, 95% CI: 52.98–66.88%) to the first visit (64.76%, 95% CI: 58.14–71.38%, *p* = 0.0435) and continued to increase until the final visit at month 3 (65.05%, 95% CI: 58.35–71.76%, *p* = 0.0116) (Fig. 6b and Data Source File 1).

Similarly, after 1 month of Collagen Activator intake, skin hydration improved. We measured cheek skin hydration in relative corneometer units (AU) and observed significant improvements throughout the study, increasing from a very dry baseline level (35.89 AU, 95% CI: 32.40–39.39 AU) to a sufficiently moistened state by the first visit (46.72 AU, 95% CI: 43.71–49.74 AU, *p* < 0.0001) and further improving by the final visit (48.32 AU, 95% CI: 45.63–51.01 AU, *p* < 0.0001) (Fig. 6c and Data Source File 1). Furthermore, forearm hydration showed significant improvement from baseline (41.96 AU, 95% CI: 39.18–44.74 AU) to the first visit (45.88 AU, 95% CI: 43.07–48.68 AU, *p* = 0.0046). However, this improvement was not maintained at the final visit (44.62 AU, 95% CI: 42.24–47.01 AU), with no significant difference observed between the baseline and final visit (*p* = 0.0792) (Data Source File 1). Remarkably, hydration of the upper arm improved significantly over time, increasing from baseline (36.02 AU, 95% CI: 33.46–38.58 AU) to the first visit (42.39 AU, 95% CI: 39.57–45.20 AU, *p* = 0.0001) and remaining elevated at the final visit (40.91 AU, 95% CI: 38.44–43.38 AU, *p* = 0.0059) (Data Source File 1). This suggests an improvement in skin hydration in multiple body parts.

Moreover, we also observed improvements in skin elasticity, as measured by the R2 viscoelasticity index on the cheek, which significantly improved throughout the study. The R2 index, expressed as a percentage, increased from the baseline (66.79%, 95% CI: 64.58–69.01%) to the final visit (70.16%, 95% CI: 67.75–72.56%, *p* = 0.0401), indicating enhanced skin elasticity (Fig. 6d and Data Source File 1).

Notably, we also observed a few sex-specific improvements upon Collagen Activator intake. For instance, significant changes were observed for brown spots within females between Baseline vs. Visit 1 (*p* = 0.0218) and Visit 1 vs. Final Visit (*p* = 0.0084; Data Source File 1). For red areas, males exhibited a significant reduction in red areas between Baseline vs. Final Visit (*p* = 0.0189; Data Source File 1).

Lastly, participants reported self-perceived improvements in their skin condition up to 6 months (Fig. 6e and Data Source File 1). Taken together, oral supplementation of Collagen Activator improved skin texture, hydration, and elasticity within 1–3 months.

### Collagen Activator reduces biological age

**Fig. 7:**
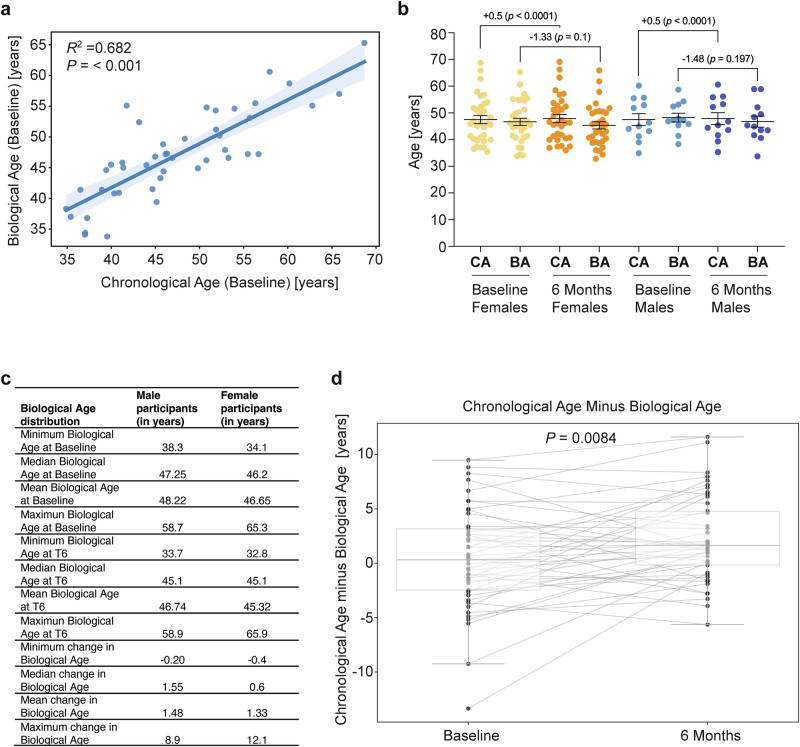
Improvements in biological age upon Collagen Activator supplementation. **a** Scatter plot depicting the relationship between chronological age and TruAge biological age at baseline of the 45 participants, with a fitted regression line and the corresponding 95% confidence interval. Adjusted *R*-squared = 0.682, *p* = < 0.001. **b** Chronological age (CA) and biological age (BA) distributions at baseline and after 6 months of Collagen Activator intake for male and female participants. **c** Gender-related effects of Collagen Activator on the biological age of study participants. **d** Chronological Age minus Biological Age difference at Baseline and 6 Months. The box plots represent the distribution of values at each time point, with individual data points overlaid as dots. Each individual’s data is connected by lines, showing the paired nature of the data. Higher values correspond to a lower biological age. Thus, individuals transitioning from a low to a higher value (Chronological Age minus Biological Age difference) represent an improvement (i.e., a reduction in biological age). A paired *t*-test revealed a statistically significant increase (*p* = 0.0084) in the Chronological Age minus Biological Age difference from Baseline to 6 Months.

At 6 months, 45 participants (33 females, 12 males) completed the saliva epigenetic test^61^ to compare it to the beginning (time point 0), before taking the Collagen Activator (Fig. 6a). At baseline, the mean chronological age with a standard deviation of 47.46 ± 8.33 was comparable to the mean biological age of 47.07 ± 7.21 years (mean difference = −0.39 years, 95% CI −1.80 to 1.03; *p* = 0.58), indicating that the participants, on average, were slightly but not statistically significant younger than expected at baseline. We performed a linear regression of biological age (dependent variable) on chronological age (independent variable). The resulting coefficient was 0.715 (95% CI: 0.565–0.865, *p* < 0.001), with an adjusted *R*-squared of 0.682. The Pearson correlation was 0.826 (*p* < 0.001) (95% CI: 0.703–0.901) (Fig. 7a).

As the natural progression of time, the chronological age increased by 0.5 years from the mean chronological age of 47.46 ± 8.33 years at baseline to 47.88 ± 8.33 years at 6 months. By contrast, the mean biological age at baseline was 47.07 ± 7.21 years, while the mean biological age at 6 months was 45.70 ± 7.57 years (mean difference: −1.37), demonstrating a statistically significant reduction (*p* = 0.040, paired *t*-test, two-tailed). When stratified by gender, the average decrease in biological age was similar between females (−1.33 years) and males (−1.48 years), with no statistically significant differences in the extent of biological age reduction between genders observed (Fig. 7b, c). The maximum decrease in biological age observed after 6 months of collagen supplementation was 8.9 years for males and 12.1 years for females (Fig. 7c).

To determine individual changes over 6 months of Collagen Activator supplementation, we plotted each participant’s chronological age minus their biological age at baseline and 6 months (Fig. 7d). A negative value indicates that the individual’s biological age exceeds their chronological age (i.e., ‘older’ biologically), whereas a positive value indicates the biological age is younger than their chronological age. For example, one participant had a baseline difference of −13.36 years, meaning their biological age was approximately 13 years older than their chronological age. After 6 months, this difference improved to −1.26, suggesting they ‘gained back’ about 12 years in 6 months. In contrast, another participant showed a +8.82 at baseline (i.e., their biological age was 8.82 years younger than their chronological age), which changed only slightly to +7.92 by the end of the study, indicating no benefit from supplementation.

This might suggest that individuals with a low biological age at baseline might not benefit as much as individuals with a high biological age at baseline. To test this idea, we compared the 25 and 75 percentiles, i.e., low vs. high biological age at baseline, and their change in biological age after 6 months of Collagen Activator supplementation. We found that individuals starting with a higher biological age than their chronological age showed the most substantial improvement in reducing their biological age after 6 months (Supplementary Fig. 5a). However, because the analysis conditions on an extreme baseline and uses a single retest, part of this pattern is expected from regression‑to‑the‑mean (RTM) rather than differential treatment response. Accordingly, we interpret this subgroup contrast as descriptive and hypothesis‑generating. To assess whether participants’ baseline biological age (BA) or their change in BA (∆BA) was related to improvements in skin parameters, we performed correlation analyses between (1) baseline BA and final-visit percentage changes in skin measures, and (2) ∆BA and final-visit percentage changes in skin measures.

Notably, baseline BA showed a positive correlation with the percentage change in cheek dermis thickness (*r* = 0.335, *p* = 0.024) but a negative correlation with the percentage change in cheek hydration (*r* = –0.298, *p* = 0.047). By contrast, ∆BA (i.e., the difference between 6-month and baseline BA) was significantly negatively correlated with forearm dermis thickness changes (*r* = −0.384, *p* = 0.009), indicating that individuals who experienced a greater reduction in BA tended to show larger increases in forearm dermis thickness. Other skin parameters demonstrated weaker or non-significant correlations. Together, these results suggest that although most skin changes were not strongly related to BA shifts, certain dermal thickness and hydration measures did show significant associations with BA or ∆BA.

Next, we wondered what else could influence the improvement from Collagen Activator supplementation. The frequency of Collagen Activator use (daily vs. every other day), exercise frequency, physical activity levels, sleep quality, and education level all showed no significant effects (Data Source File 1). Although 66% of participants use other supplements, we did not find any significant difference in the biological age of participants who had taken other supplements vs. non-supplement users (Supplementary Fig. 5b), suggesting that the improvements observed with the Collagen Activator are independent of or synergistic with other supplements. By contrast, individuals who consumed more than one drink per week had fewer improvements with the Collagen Activator than individuals who did not drink any alcohol (Supplementary Fig. 5c), suggesting a less favorable interaction between the Collagen Activator and alcohol consumption. Thus, after 6 months of using the Collagen Activator in this clinical study, we observed, on average, a 17-month reduction in biological age.

## Discussion

Collagen supplementation in *C. elegans*^32,33^, *Drosophila*^31^, and rats^30^ increases their health and lifespan^38,62^. In humans, clinical studies showed the benefits of collagen supplementation for skin, muscle strength, tendons, nails, joint pain, glucose, and lipid metabolism^9^^–^^15,27^^–^^29^. However, how and which part of collagen leads to these physiological improvements is unknown. Here, we showed that a simple ratio of three amino acids, 3 glycine—1 proline—1 hydroxyproline, was sufficient to counteract the age-related gradual decline of collagen and increase the healthspan and lifespan of *C. elegans*. Supplementing human skin cells with [3 Gly : 1 Pro : 1 Hyp] increased collagen gene and other ECM remodelers within hours, suggesting a universal and conserved recognition signal elicited by [3 Gly : 1 Pro : 1 Hyp] supplementation. Moreover, [3 Gly : 1 Pro : 1 Hyp] supplementation in very old mice was safe and counteracted the age-related increase in fat and loss of muscle. Given the additive longevity effect of [3 Gly : 1 Pro : 1 Hyp] supplementation with alpha-ketoglutarate and the fact that alpha-ketoglutarate is a cofactor for 4-prolyl hydroxylase, an enzyme that stabilizes collagens, we generated a combined formulation, including vitamin C and astaxanthin, both of which improve collagen homeostasis, before going into humans. We found that this Collagen Activator was safe and effective for improving dermatological features and reducing participants’ biological age.

Several benefits of these three amino acids have been attributed to being at the nexus of nutrition, metabolism, and healthy aging. Proline serves as a major amino acid and nitrogenous donor for synthesizing polyamines, which are key regulators of DNA and protein synthesis^63^. Increasing endogenous proline levels increases the replicative lifespan of yeast^64^. Similarly, exogenous proline supplementation restores mitochondrial function in senescent human mesenchymal stem cells and increases the lifespan of *C. elegans* through mitochondrial catabolism^65,66^. This proline-induced longevity depended on *mev-1*/succinate dehydrogenase of complex II in the mitochondrial electron transport chain, energy-sensing *aak-2/*AMP-activated protein kinase that is upstream of mTORC1 and *rsks-1*/Ribosomal protein S6 kinase and key transcription factors *daf-16/Foxo* and *skn-1/Nrf1,2,3* that are downstream of mTORC1, a key regulator of nutrient sensing and growth^42,67^. By contrast, proline-induced longevity did not require amino acid sensor *gcn-2* nor *gas-1* in mitochondrial complex I that is required for oxidative phosphorylation^42^, suggesting proline supplementation increases lifespan via distinct genetic pathways involved in nutrient sensing, growth, and mitochondrial metabolism of *C. elegans*.

Although reports on supplementing just hydroxyproline for healthspan or lifespan are missing, in fish and shrimp, adding hydroxyproline to the diet increases muscle growth and stimulates collagen gene expression^68–70^. In mice models of inflammatory bowel disease, hydroxyproline supplementation inhibited NF-kB inflammatory signaling and improved oxidative stress response^71^. In humans, hydroxyproline can not be reincorporated into collagen, but it is metabolized by mitochondria and converted to glycine by the peroxisome^4,72^. The physiological effects of oral intake of hydroxyproline, directly on health or indirectly by converting it into glycine, need further research.

By contrast, glycine supplementation has been documented with several healthspan and lifespan improvements. Glycine supplementation increases the lifespan of *C. elegans*^41,42^, Fisher 344 rats^73^, and genetically heterogeneous UM-HET3 mice^74^. In humans, glycine levels in the blood circulation decline with age in old men^75^. Furthermore, genetic variants in humans that raise glycine in blood show a reduced risk for cardiovascular diseases^75^. Moreover, higher glycine levels in the blood are associated with improved insulin sensitivity^76^ and reduced risk of diabetes^77^, suggesting improved metabolic health.

Consistent with our observation of Collagen Activator supplementation slowing fat accumulation and improving grip strength in old mice, glycine supplementation in sucrose-fed rats prevented abdominal fat accumulation and lowered blood pressure^78^. Extracellular glycine is essential for the proliferation of muscle progenitor cells in old mice, and it has been shown in vitro using primary human muscle progenitor cells^79^. Furthermore, glycine supplementation during caloric restriction prevented muscle loss and accelerated fat loss in mice fed high-fat diets^80^.

The underlying mechanisms elicited by glycine supplementation are unclear on a molecular level. In lean mice, glycine supplementation suppresses TNFalpha, shows anti-inflammatory effects, and prevents insulin resistance in obese mice^81^. Glycine administration was neuroprotective by regulating the c-Jun N-terminal kinase in neurodegenerative disease mouse models^82^. Glycine supplementation in old mice increases mitochondrial biogenesis^83^. In *C. elegans*, glycine supplementation increases collagen mRNA^41^, consistent with our findings with [3 Gly : 1 Pro : 1 Hyp] supplementation. However, this glycine-induced longevity was shown to be mediated via the methionine cycle and one-carbon metabolism^41^. These findings suggest that glycine supplementation might work through one-carbon and mitochondria metabolism-regulating stress and inflammatory responses in preclinical models.

There is evidence that these findings in preclinical models are conserved in humans. Supplementing glycine to human patients with metabolic disorders protects against oxidative stress and inflammation^84,85^. In a systematic review analyzing 34 randomized controlled trials, glycine supplementation had the most positive effects on the nervous system, improving sleep quality and cognition in healthy adults^86^. Reassuringly, for a potential direct effect, oral supplementation of glycine increased glycine levels in the brain in men^87^. In the brain, glycine acts as an inhibitory neurotransmitter^88^. Increasing glycine levels in the suprachiasmatic nucleus in the brain binds NMDA receptors, inducing hypothermia and sleep^89^. In addition, in diseased individuals, glycine supplementation improved glucose metabolism by increasing insulin response and lowering A1C and HOMA-IR^86^. Consistent with our finding of feeding Collagen Activator or glycine in old mice from other labs, glycine supplementation in humans promoted increased lean mass and handgrip strength, as assessed by the systematic review^86^.

Therefore, there is a body of increasing evidence that administering these three amino acids promotes health in preclinical models and humans. Administering our novel and patented combination of a more collagen-mimicking ratio [3 Gly : 1 Pro : 1 Hyp] as a tool compound in preclinical models and then optimization by adding supporting ingredients (alpha-ketoglutarate, astaxanthin, and vitamin C) showed improvements in skin features and reduced biological age by 1.33 years in women and 1.48 years in men (pooled mean difference −1.37; *p* = 0.04). Although in our study, 3 months of Collagen Activator supplementation did not significantly improve wrinkles, other larger randomized, double-blinded, placebo-controlled clinical trials showed that collagen peptide supplementation improved wrinkles within 2–3 months^9,10^. Nevertheless, we demonstrated that 3 months of oral supplementation of Collagen Activator improved skin elasticity, hydration, and texture, consistent with several larger randomized, double-blinded, placebo-controlled clinical trials using collagen peptides^9–12^. Given that we used oral supplementation, the improvements in skin features must come from within the body. We confirmed this by assessing systemic biological age using saliva.

Epigenetic methylation clocks, which reflect biological aging, provide a valuable tool to assess the effectiveness of interventions aimed at reducing biological age^90–92^. A prior study utilized the TruMe epigenetic saliva test, similar to our study’s assessment method. The article details a research investigation into the effects of Rejuvant®, a formulation consisting of alpha-ketoglutarate paired with vitamin D for women or vitamin A for men, on biological aging. This study involved 42 healthy participants, with an average chronological age of 63 years. Over an average period of 7 months, participants experienced a significant reduction in biological age by an average of 8 years, as measured by the TruAge DNA methylation test. The results indicated notable decreases in biological age for both genders, with the treatment being most beneficial for individuals whose biological age was higher than their chronological age. Nonetheless, as the study was not placebo-controlled, further research is warranted to confirm these findings^61^. Supplementation of a combination of vitamin B3, vitamin C, vitamin D, Omega 3 fish oils, resveratrol, olive fruit phenols, and similarly astaxanthin for 3 months did not significantly reduce biological age based on DNA methylation, applying the Horvath clock in a cohort with an average age of 72 years^93^. However, in that study, participants who already had a very high biological age at baseline based on the InflammAge clock showed a reduction in biological age^93^, suggesting that the older their biological age, the larger the gap between chronological age and biological age at baseline, the more the participants benefited in reducing biological age. Similarly, we also observed in our study that participants with an older biological age showed the most reduction in biological age, on average, a 5-year reduction but up to 13 years of improvements during the 6 months of Collagen Activator supplementation (Supplementary Fig. 5a). In a randomized clinical trial with obese African Americans having low vitamin D at baseline, supplementation of vitamin D for 4 months decreased biological age by 1.9 years based on the Horvath and Hannun clocks compared to the placebo-controlled group^94^. In one of the most extensive clinical studies across Europe (DO-HEALTH), supplementation of vitamin D, omega-3, and home-based exercise reduced prefrailty by 39%^95^ and incident invasive cancer by 61%^96^ over a 3-year follow-up. From this study, 777 participants, with an average age of 75 years, who underwent 3 years of supplementation of vitamin D, omega-3, and home-based exercise showed a reduction in biological age by 3–4 months^97^. Dietary restriction is one of the most potent interventions to increase life- and healthspan in pre-clinical models. In the CALERIE trial, a 2-year randomized controlled multicenter phase 2 clinical trial with 220 adults (age 20–50 years), a 12–25% reduction in calorie intake did not reduce biological age but slowed the pace of aging using the DunedinPACE DNAm algorithm^98^. Similarly, less pronounced effects were observed in a randomized clinical study with 219 women (aged 50–69 years; DAMA trial), a 2-year dietary intervention with plant-rich food but not higher physical activity, reduced biological age by 8 months using the GrimAge^99^. In 43 healthy men (aged 50–72 years), a 2-month intervention of a combination of diet, sleep, exercise, relaxation guidance, and supplemental probiotics and phytonutrients reduced biological age by 2 years using the Horvath clock^99^. These clinical trials suggest that lifestyle, dietary, and supplement interventions can reduce biological aging by a few months to a few years.

In comparison to these previous trials, we conducted an observational study to evaluate the safety, benefits, and potential of the Collagen Activator in reducing biological age and improving skin quality among healthy individuals aged 35 years and above. In our study, the average age was 47 years, which is before or at the tipping point where signs of aging start to appear. Participants in our study were considerably younger than other previous cohorts using epigenetic age clocks. Furthermore, our participants were in excellent physical shape and good health. Most of them had a university degree and lived a healthy lifestyle (little or no alcohol, vegetarianism/flexitarianism, not smoking, and plenty of exercise). By measuring DNA methylation patterns and biological age, our trial also provides an opportunity to further establish the correlation between epigenetic clocks and relevant health outcomes, specifically multiple measures of skin aging. As validated in previous clinical trials, components of the Collagen Activator, including glycine, collagen peptides, alpha-ketoglutarate, and astaxanthin, were well-tolerated and safe, and some have been previously shown to be effective in reducing biological age in humans. Given this younger and healthier cohort, it is remarkable to observe, on average, a reduction of 1.4 years of biological age within 6 months of Collagen Activator supplementation.

To what extent the [3 Gly : 1 Pro : 1 Hyp] supplementation alone drives the reduction in biological aging in humans can only be speculated upon. A further limitation of this study is that it was not placebo-controlled, which might be a future research study designed as a randomized, double-blinded, placebo-controlled study that also included more elderly people above the age of 75 years. Further future studies might also focus on revealing the underlying mechanisms of how cells and tissues read out the 3 glycine to 1 proline to 1 hydroxyproline ratio as an AND-gate function.

Our study presents promising evidence for the health and aging benefits of a collagen-derived amino acid composition across multiple model systems. Still, several critical limitations need to be acknowledged to contextualize the findings and guide future research. Although past literature shows that individual amino acids like glycine and proline can extend lifespan in *C. elegans*, in the main survival assays, the benefit of the 3 Gly : 1 Pro : 1 Hyp mixture was not compared directly with equivalent molar doses of native collagen or other amino acid combinations outside of the reported motility assays. This was due to osmolarity challenges in culturing NGN agar plates, resulting from trying to establish equivalent molar doses. Furthermore, not all possible combinations were tested due to practical constraints, and a complete mechanistic understanding of cellular “ratio sensing” remains to be determined in future research. The mouse experiments did not include isonitrogenous or amino acid-matched controls, posing a potential risk for amino acid imbalance. However, total amino acid intake must be modified to a much greater extent to elicit a robust physiologic effect than we used in our mouse study with a standard control diet and the 3 Gly: 1 Pro: 1 Hyp formulation at modest doses, modifying overall protein intake by approximately 5%. Lastly, the human trial was open-label, single-arm, and observational rather than randomized and placebo-controlled. All participants consumed the collagen activator, so placebo effects, regression to the mean, and selection bias cannot be excluded. Furthermore, the greater improvement observed among participants whose biological age exceeded chronological age at baseline (Supplementary Fig. 5a) is susceptible to regression-to-the-mean (RTM). In pre–post designs that stratify on an extreme baseline and assess change with the same assay, random within-person variability and measurement error naturally drive follow-up values toward the group average, potentially exaggerating apparent benefits in the “high BA” subgroup, even in the absence of an actual effect. Our trial was open‑label and single‑arm; therefore, we cannot disentangle RTM from a genuine effect modification by baseline status. Future studies should employ randomized, placebo-controlled designs with isonitrogenous and single-amino-acid control arms, both in animal models and humans.

In summary, it is remarkable that the physiological and molecular effects of combining these three amino acids are conserved across *C. elegans*, mice, human fibroblasts, and human participants. We demonstrated that upon [3 Gly : 1 Pro : 1 Hyp] supplementation, collagen and ECM homeostasis were elicited during the old age of *C. elegans*, in human cell culture, and visible in dermatological improvements in human skin. Similarly, we showed that [3 Gly : 1 Pro : 1 Hyp] supplementation increased the healthspan and lifespan of *C. elegans*, counteracted age-related strength loss and obesity in old mice, and reduced biological age in humans. This suggests an ancient underlying mechanism for detecting the ratio of these three amino acids.

## Methods

### *C. elegans* strains

*Caenorhabditis elegans* strains were maintained on NGM plates and OP50 *Escherichia coli* bacteria. The wild-type strain was N2 Bristol. Mutant strains used are described at www.wormbase.org: TJ1060 *spe-9(hc88)* I; *rrf-3(b26)* II., JK2729 *dpy-18(ok162)* III. Transgenic strain LSD2002 *spe-9(hc88)* I; *xchIs001* [P*col-144*:: GFP; *pha-1*(+)] X^38^.

### Supplementation of amino acids for collagens reporter readout in *C. elegans*

For determining the most effective amino acid ratios (Figs. 1a–e and S1c–f) 0.1 M stocks of glycine (Sigma-G7126), proline (Sigma-P5607), and hydroxyproline (Sigma-56250) were prepared in filter-sterilized via Millipore 0.2 μm pore membrane (SARSTEDT-83.1826.001) mQ-H2O. Afterward, these stocks were added into NGM agar during preparation (with either carbenicillin or ampicillin and nystatin; see WormBook nematode maintenance protocols), and mixed thoroughly prior to solidifying. Thus, corresponding amino acid concentrations were achieved: either 50 μM, 500 μM, or 5000 μM. Finally, 5 mM corresponds to “one ratio point” later (3-1-1 = 15 mM glycine – 5 mM proline – 5 mM hydroxyproline). After determining 1 ratio point, all further experiments were done in the same way, except for stocks, where amino acids were solubilized in mQ-H2O to their maximal solubility points: 4 M for glycine, 3.17 M for proline, and 198 mM for hydroxyproline, correspondingly. This was done to decrease excessive dilution of NGM with water, especially in experiments with 10-1-1, 1-1-2, and 1-1-3 ratios, to avoid high salt imbalance in nematode growth media. Control and amino acid-containing plates were kept at room temperature for 2–3 days to dry after preparation. Fresh OP50 bacteria were grown a day before the experiment to achieve their stationary phase (8 h or overnight). Then, it was heat-inactivated (to exclude metabolizing amino acids by bacterial cells) at 60 °C for a minimum of 45 min and cooled down to RT. Then, it was concentrated 10-fold with 2809 rcf/15’ centrifugation and seeded onto plates after resuspending the bacterial pellet. One ml, 300 μl, or 120 μl of bacterial concentrate were added to 15 cm, 6 cm, or 3.5 cm plates, respectively. After seeding, the plates were ready to use once the NGM absorbed all the excess liquid. The quantification was performed according to Statzer et al.^38^.

### Lifespan assays

Manual lifespan assays were performed at 20 °C on heat-inactivated OP50 bacteria, seeded onto NGM plates containing nystatin if otherwise stated. N2 and JK2729 worms were grown until the L4 stage on plates with heat-inactivated OP50, then transferred to 50 μM FUdR-containing plates to prevent progeny development. On the other hand, TJ1060 and LSD2002 worms were kept at 25 °C until the young adulthood stage, the restrictive temperature for *spe-9(hc88)* temperature-sensitive mutation, and then, after confirmation of the *C. elegans’* sterility, re-picked to plates with corresponding conditions, as stated in the experiments. *C. elegans* were observed/counted every second day until day 12 of adulthood (AD12), with daily quantification later on until the end of the experiment. All animals that dried on the plate walls deeply dug into agar, as well as *C. elegans* with protruding vulva and matricide phenotype, were documented as censored for further analysis. Manual lifespan data were calculated using the Kaplan–Meier estimator in GraphPad Prism™ 8.0.1 software. Log-rank (Mantel–Cox) statistical analysis was used to determine the significance. Mean lifespan changes were calculated separately with a *t*-test or One-way ANOVA and SEM (if not stated otherwise), utilization for statistics in the same software package.

### Longitudinal movement assays

First, the population of 2000–5000 *C. elegans* were synchronized at day 1 to day 4 of adulthood (AD1-AD4) and bleached^100^. One μl of ampicillin (100 mg/mL) and 0.5 μl of tetracycline (100 mg/mL) per 1 ml of buffer with bleached animals were added. The animals were left overnight for hatching and L1-synchronization. Second, the synchronous L1 populations of *C. elegans* were filtered via an 11.0 μm pore size, hydrophilic nylon membrane (Millipore—NY1104700). The clean population of L1s was counted under a stereomicroscope in 3 ×10 μl drops to define *C. elegans* concentration. Meanwhile, 50 ml of stationary-phase fresh heat-inactivated OP50 bacteria were concentrated, and all LB-media leftovers were discarded. Then, the bacterial pellet was resuspended thoroughly in 10 ml of S-complete buffer in aseptic conditions <10 cm from a gas burner. Before the experimental procedure, all the pipettes and pipette boys were sprayed with 70% ethanol. A total of 3000–4000 animals from the filtered L1 populations were concentrated at 1800 rcf/1’ in 15 ml Falcon®, and M9 buffer was discarded until almost a packed worm pellet was reached. Afterward, 10 ml of S-complete with food was added to *C. elegans* and well-mixed. Later, this tube was sprayed with 70% ethanol, transferred under a sterile laminar-flow hood, and poured into a 10 cm sterilized tray. Using a multichannel pipette, 100 μl of buffer with *C. elegans* was resuspended and added into the U-bottom 96-well plate per well, achieving ~ 30–40 animals per well. Plates with worms were left in small sealed plastic bags at 20 °C (or 25 °C for TJ1060) on a working orbital shaker (450–500 rpm) for internal aeration. Animals were checked every day for the presence of contamination and abnormalities in growth and development. After reaching the L4 stage, 100 μM FUdR of final concentration was added for N2 *C. elegans*. Corresponding amino acids were added at AD1 in the mentioned concentrations. Animals were fed with additional heat-inactivated bacteria (but 50% of the initial amount) every 5th day until adulthood day 15 (AD15), when they drastically dropped food consumption. Motility (fitness) measurement was performed using WMicrotracker ONE ® (from InVivo Biosystems™). We used 3 ×30 min “time-buckets” for measurements, and the mean value was calculated for each well. In turn, we utilized eight wells per condition for robust quantification. XY-graphs (health-span curves) were built in GraphPad Prism™ 8.0.1 software for absolute motility readings. The area under the curve (AUC) for descriptive statistics and the two-way ANOVA test were used to calculate the significance between curves, and mean values of 8 wells of the same condition were used as reference points.

### Cultivation and 3 Gly : 1 Pro : 1 Hyp supplementation of human dermal fibroblasts

Human dermal fibroblasts (HDF, neonatal, Sigma-Aldrich, SKU 106-05N) were grown in a fibroblast growth medium (Sigma-Aldrich, SKU 116-500). Before 3-1-1 supplementation, cells were harvested using accutase cell detachment solution (w: 0.5 mM EDTA, w: Phenol red; Pan-Biotech; P10-21500) and centrifuged (200 × *g*, 5 min, RT). Cells were resuspended in fibroblast growth medium without supplementation or with a mixture of 3 mM glycine, 1 mM proline, and 1 mM hydroxyproline (sterile filtered). Subsequently, HDFs were seeded in 10 cm plates (30% confluency). Cells were cultivated for 2 h, 8 h, and 24 h and lysed on the plate using 1 ml TRIzol-Reagent (ambion®, ref# 15596018). Cells harvested at 0 h were centrifuged again (200 × *g*, 5 min, RT) immediately after resuspension and lysed using 1 ml Trizol. RNA extraction was performed using a standard phenol-chloroform method. Briefly, 1 mL Trizol lysate was combined with 0.2 mL chloroform, mixed, and centrifuged (10,000 × *g*, 10 min, 4 C). The upper layer (~0.4 mL) was taken, combined with 0.5 mL of isopropyl alcohol, mixed, and centrifuged (10,000 × *g*, 10 min, 4 C). The supernatant was discarded, and the pellet was washed twice with 80% ethanol, dried, and resuspended in RNase-free water.

### RNA sequencing

RNA quality was confirmed using a NanoDrop 1000 spectrophotometer and an Agilent 2100 Bioanalyzer. cDNA libraries were prepared using the Illumina TruSeq Stranded Total RNA Sample Preparation protocol. A total of 150 bp paired-end reads were generated on an Illumina NovaSeq with a read depth of 20 million paired reads per sample. Reads were aligned and annotated to the Ensembl *Homo sapiens* GRC38.p13 reference genome and associated GTF annotation file using the align and featureCounts functions from the Rsubread package (2.10.4) in R (4.2.0). Differential gene expression analysis was performed using the voom and eBayes functions from the EdgeR (3.38.1) and Limma (3.52.2) packages. Gene set enrichment analysis was performed using the gseGO function from the clusterProfiler package (4.4.4). RNA-seq data can be found in the NIH SRA database under the accession number PRJNA856884.

### Mouse study

Male C57BL/6J mice were ordered from Janvier between the ages of 18 and 21 months, equivalent to approximately 55 to 60 human years of age. All ordered mice were included in the study except for the ones that reached termination criteria before the start of the study, as defined in the animal license. Mice were allowed to acclimate to the facility for approximately 1 month before baseline measures were taken and experimental diets were initiated. The Swiss Federal Food Safety and Veterinary Office approved the study, and the animal license ZH149/21 was accepted via animex-ch (https://www.animex-ch.blv.admin.ch/).

### Mice, husbandry, and diets

The animal rooms were kept at 20–22 °C and 50–70% humidity. The mice were held in individually ventilated cages with a 12:12 h light-dark cycle, with the dark period occurring from 8 a.m. to 8 p.m. All measurements were performed during the dark period. Up to four mice were housed in one cage with ad libitum access to food and autoclaved drinking water. All animals were provided enrichment, including houses, nestlets, and chew blocks. Mice cages (including water bottles and enrichment) were changed weekly in a laminar flow hood, and body weight and food intake were recorded for all mice. Mice underwent daily monitoring during the study according to the parameters set out in Appendix 4. Mice reaching humane endpoint termination criteria were euthanized according to approved ethical protocols. Before starting the study, all mice were fed a standard AIN-93G-based diet for up to 1 month, during which time the baseline measurements were performed. A mixture of 3:1:1 ratio glycine:proline:hydroxyproline was mixed into the diet at 334 mg/kg of diet to achieve a final dose to mice of approximately 100 mg/kg body weight. All study diets were obtained from a commercial vendor (Research Diets, New Brunswick, NJ). After baseline measurements, mice were given either a control or 3:1:1 diet for 6 months. Cages were randomly assigned to a diet group. At the end of the study period, mice were euthanized by carbon dioxide inhalation, and tissues were collected for weighing and analysis. Organ weights were recorded for liver, kidney, heart, epididymal adipose tissue, interscapular brown adipose tissue, and spleen (Data Source File 1). There were no significant differences in organ weights other than the epididymal adipose tissue (Data Source File 1). A gross necropsy was performed to note macroscopically visible pathologies, e.g., tumors, cirrhosis, etc. No significant group differences were identified in these assessments.

### Mouse clinical frailty index (mFI)

The mFI is a tool to quantify general health in mice over several organ systems, which has previously been shown to correlate with mortality risk. To perform the mFI, all mice were assessed non-invasively for the presence or absence of a total of 33 health parameters, and the severity for each was graded as 0, 0.5, or 1. The original mFI developed by Whitehead et al.^101,102^ includes 31 parameters, two of which were added in this study: the ledge test^103^ and hind limb clasping. For the ledge test, the mice were evaluated for their ability to climb on a grid, which is a direct measure of coordination that can be impaired in ataxias and neurodegenerative diseases. Hind limb clasping is a phenotype associated with neurological dysfunction, which was observed in this study’s aged mice and, therefore, incorporated into the mFI. The clasping was assessed by suspending the mice by the tail and scoring the degree by which the hind limbs were clasped towards the body. The ledge test and hind limb clasping were graded as 0, 0.33, 0.66, and 1. To calculate the mFI, the scores for each parameter were added up, and the sum was divided by 33 to get a value between 0 and 1, which allows a straightforward interpretation of the mFI. For more details on the scoring system, see Appendices 1–3.

### Collagen Activator formulation

One sachet of Collagen Activator contains active ingredients: patented 8400 mg Colgevity™ (5000 mg L-Glycine (Xi’an SLT Biotech Co., Ltd., Chemical synthesis), 1700 mg L-Proline (Xi’an SLT Biotech Co., Ltd., Corn fermentation), 1700 mg L-Hydroxyproline (Xi’an SLT Biotech Co., Ltd., Glucose Fermentation)), 1000 mg calcium alpha-ketoglutarate (Botanic Healthcare Pvt Ltd., Chemical synthesis), 200 mg algae powder (providing 4 mg astaxanthin; Botanic Healthcare Pvt Ltd., Chemical synthesis), and 150 mg acerola fruit juice extract (providing 48 mg vitamin C; Vidya Herbs Pvt. Ltd., Acerola fruit juice extraction).

Supporting ingredients were selected for their functional properties. Silicon dioxide was included as an anti-caking agent to ensure the powder’s free-flowing nature, improve manufacturing processes, maintain ingredient uniformity, and enhance the stability of the final product. Citric acid was added as an acidity regulator to stabilize pH, enhance the flavor, improve solubility, prevent microbial growth, and protect the product’s active ingredients, ensuring a high-quality and enjoyable final product. Steviol glycosides derived from stevia serve as a natural, calorie-free sweetener to enhance the taste, mask unpleasant flavors, and improve palatability.

The ingredients were sourced globally for quality control, focusing on high bioavailability and activity. Every batch underwent rigorous double-testing for purity by an independent Swiss lab (Data Source File 1).

### Clinical trial design

Swissethics (Kantonale Ethikkommission Zürich) granted ethical approval for the study (BASEC ID number 2023-00953). The clinical observational trial was registered at ISRCTN registry. Clinical trial registry name: Reverse the Clock: A Clinical Trial of Collagen Activator Effects on Biological Age and Skin Quality. Registration number: ISRCTN93189645. Date of registration: 03/07/2025. CONSORT checklist provided in Supplementary Information. Written informed consent was obtained from all participants after a full explanation of the study. An observational human trial was conducted, with 66 subjects recruited and informed to consume one sachet (powder) of Collagen Activator daily for 6 months. Inclusion criteria: (1) Healthy adults aged above 35 years old, regardless of gender, ethnicity, or socioeconomic background; (2) Voluntarily purchasing the collagen activator (from Avea) for 6 months with the intent to take it daily (3) able to commit to 3 visits at the dermatology clinic Hautwerk in Zürich. The exclusion criteria included: (1) chronic skin conditions; (2) chronic medical conditions, including diabetes, cardiovascular diseases, kidney disease, liver disease, gastrointestinal disease, cancer, and autoimmune diseases; (3) pregnant and breastfeeding; (4) taking chronic medications; (5) people who had any cosmetic procedures (intense pulse light, medical peelings, laser therapy, microneedling, Botox, fillers) before 3 months of the study; (6) people who had taken other collagen supplements 3 months before the first screening date; (7) insufficient knowledge of the German or English language. Participants were recruited through a dedicated landing page on Avea Life website, newsletters, word of mouth, social media channels, and recruitment materials distributed at relevant conferences. Each participant underwent three skin measurements at the baseline, after 4 weeks, and after 12 weeks, respectively. Before each skin measurement, subjects were instructed to refrain from washing their face for 6 h, use any skin care products for 12 h, and acclimatize for 10 min before the visit. Self-assessment questionnaires of the subjects were collected monthly for 6 months. The biological age was measured via an epigenetic methylation clock test. Participants were asked to take the test via saliva collection at the baseline, and after 6 months, they began taking the Collagen Activator.

Study data were collected and managed using REDCap electronic data capture tools hosted at ETH Zurich^104^. REDCap (Research Electronic Data Capture) is a secure, web-based software platform designed to support data capture for research studies, providing (1) an intuitive interface for validated data capture; (2) audit trails for tracking data manipulation and export procedures; (3) automated export procedures for seamless data downloads to common statistical packages; and (4) procedures for data integration and interoperability with external sources.

### Skin measurements

A Visia Cam (Canfield Scientific Inc.) was used to capture in-depth insights into skin conditions. The system is based on Canfield’s RBX® technology, which utilizes cross-polarized light and UV illumination to assess reference values for spots, wrinkles, texture, pores, UV spots, red areas, and porphyrins. After image acquisition, specialized software processes the data using algorithms that detect and quantify skin parameters by identifying image patterns. The results are then compared to a comprehensive database of individuals grouped by demographic factors such as gender, age, and skin type. This database enables comparative analysis, with the software calculating a percentile for each parameter, positioning the patient’s skin condition relative to the corresponding demographic group.

A Corneometer CM 825 (Courage and Khazaka, Cologne, Germany) was used to assess skin hydration of the cheek, forearm, and upper arm. Utilizing a capacitive measurement principle, the Corneometer quantifies the moisture content of the stratum corneum by detecting changes in capacitance that occur due to water’s dielectric properties. Three measurements were performed for each location, and the values were averaged.

A Cutometer MPA 580 (Courage and Khazaka) was used to assess skin elasticity of the cheek, forearm, and upper arm, using a 2 mm probe in Mode 1. The device operates based on the suction method, where negative pressure is applied to the skin, and the resulting deformation is measured to evaluate the biomechanical properties of the skin. For each measurement, a target pressure of 450 mBar was applied, with an on-time of 3 s and an off-time of 3 s, allowing the skin to recover between cycles. Five repetitions were performed at each measurement site to ensure reliability and consistency in the results. The R2 value, representing gross elasticity, assessed the skin’s overall elasticity. This parameter reflects the skin’s ability to return to its original shape after deformation, combining immediate and delayed elastic recovery.

Measurements with both the Corneometer and the Cutometer were conducted on three standardized areas: on the left cheek, at a point 5 cm from the mouth corner along the line connecting the mouth corner to the tragus, at the midpoint of the right volar forearm, and the midpoint of the medial side of the right upper arm.

The measurements were taken under controlled environmental conditions of 22 °C and 45% humidity, with no daylight or windows in the room to ensure consistent conditions. Additionally, participants were instructed to refrain from contact with water and the use of cosmetics before the measurements (6 h and 12 h, respectively) to eliminate any external factors that could influence the results.

### DNA sample collection and DNA methylation calculation

Saliva samples were taken twice: at the baseline, they were collected at Hautwerk dermatology clinic under the doctor’s supervision. After 6 months, participants collected them at home using commercially available TruMe sampling kits. After collection, the samples were shipped to TruMe Labs for analysis. DNA methylation and DNAm age have been calculated, as previously reported by Demidenko et al.^61^. In particular, the TruMe TruAge epigenetic clock is a DNA methylation-based assay designed to estimate biological age by analyzing methylation patterns at nine specific CpG sites. Saliva-derived DNA samples were analyzed using locus-specific Sanger sequencing. The TruAge™ test, an epigenetic clock developed by TruMe Inc., estimates an individual’s biological age by quantifying DNA methylation (DNAm) levels at selected CpG loci within the genome. Although the specific CpG identifiers (csIDs) and the complete algorithmic formula remain proprietary, the test operates on a well-established principle: a weighted mathematical combination of methylation measurements at preselected genomic sites known to correlate with aging.

The initial development of the TruAge clock involved screening over 900 CpG sites identified from publicly available DNA methylation (DNAm) datasets, chosen for their age-related variability and biological relevance. Candidate markers were filtered based on (1) above-median variation in beta values and (2) strong correlation between methylation status and chronological age (*R*² ranging from 0.38 to 0.95). From this pool, nine CpG sites were selected for locus-specific validation based on their high predictive value and genomic localization within regulatory regions of aging-associated genes. These sites exhibited methylation changes up to 40% across the human lifespan. A broader TruAge™ Index algorithm was also constructed using a panel of approximately 33 CpG sites, with the validated nine forming the foundation of the core predictive model. The TruAge™ clock estimates biological age via a regression-based formula:

1 $$\mathrm{Biological}\,\mathrm{Age}=C+\mathop{\sum }\limits_{i=1}^{n}{w}_{i\,}\times {M}_{i}$$

Where:

- *C* is a model intercept (constant),
- *w*₁ … *w*_*n*_ are site-specific weights (regression coefficients),
- *M*₁ … *M*_*n*_ are the beta values of DNA methylation at each CpG site, and
- *n* is the number of CpG sites used in the model (nine in the validated TruAge™ test).

Weights were derived using statistical learning methods, including multivariable regression and machine learning, trained to optimize age prediction accuracy.

TruAge™ utilizes saliva samples as the input biospecimen. Saliva was selected due to its non-invasive collection, 70% DNAm concordance with blood, and logistical convenience for at-home self-sampling protocols. Methylation quantification is performed using Sanger sequencing or pyrosequencing, both of which are standard high-resolution methods for detecting DNA methylation. The TruAge™ model was initially validated in a cohort of 105 healthy adults, achieving a Mean Absolute Deviation (MAD) of 4.67 years in chronological age prediction. Subsequent refinement of the model, incorporating data from over 1000 clinical trial participants (TruMe customer database; results pending publication), improved predictive performance to a MAD of 3.8 years. To assess analytical validity, TruAge™ predictions were compared against the MyDNAge® clock (ZymoGenomics), demonstrating strong agreement with a correlation coefficient (*R*² = 0.95, unpublished). Beyond correlation with chronological age, the TruAge test has demonstrated sensitivity to biologically meaningful changes in aging status. While established epigenetic clocks—such as Horvath’s pan-tissue clock, Hannum’s DNAm age, Levine’s PhenoAge, GrimAge, and DunedinPACE—remain key benchmarks in the field, the TruAge Index was selected for this study due to its validated performance in saliva samples, ease of use, and robust responsiveness to biological age-modifying interventions. Its strong predictive capacity and concordance with established biomarkers of aging make it a suitable tool for our cohort and study objectives.

### Self-reported questionnaires

Online questionnaires were sent monthly to assess product intake frequency and overall health and skin conditions.

### Statistical analysis

Shapiro–Wilk test was used to assess data normality. Repeated measures ANOVA or the Friedman test was applied to evaluate changes over time. For comparisons, paired *t*‑tests (or Wilcoxon signed‑rank tests) were performed for matched data, and unpaired *t*‑tests (or Mann–Whitney *U* tests) were performed for independent samples. Linear regression and Pearson correlation examined associations between baseline chronological and biological age. A chi-square test was employed to evaluate associations between categorical variables. All analyses were conducted in GraphPad Prism and R, and statistical significance was set at *p* < 0.05.

## Supplementary information

Supplementary Information

Supplementary Table

Supplementary Table

Supplementary Table

Supplementary Table

Supplementary checklist

Source Data

## Data Availability

RNA-seq data can be found in the NIH SRA database under the accession number PRJNA856884. Data are provided within the manuscript or Supplementary Tables 1–4 and Data Source file 1.
