# Supplementary material for: A collagen amino acid composition supplementation reduces biological age in humans and increases health and lifespan in vivo
Source: NPJ Aging. 2025 Nov 20;11(1):91. doi: 10.1038/s41514-025-00280-7 (PMC12635253; doi:10.1038/s41514-025-00280-7)
Supplement: Supplementary file 1 — Supplementary Information [file 41514_2025_280_MOESM1_ESM.pdf]

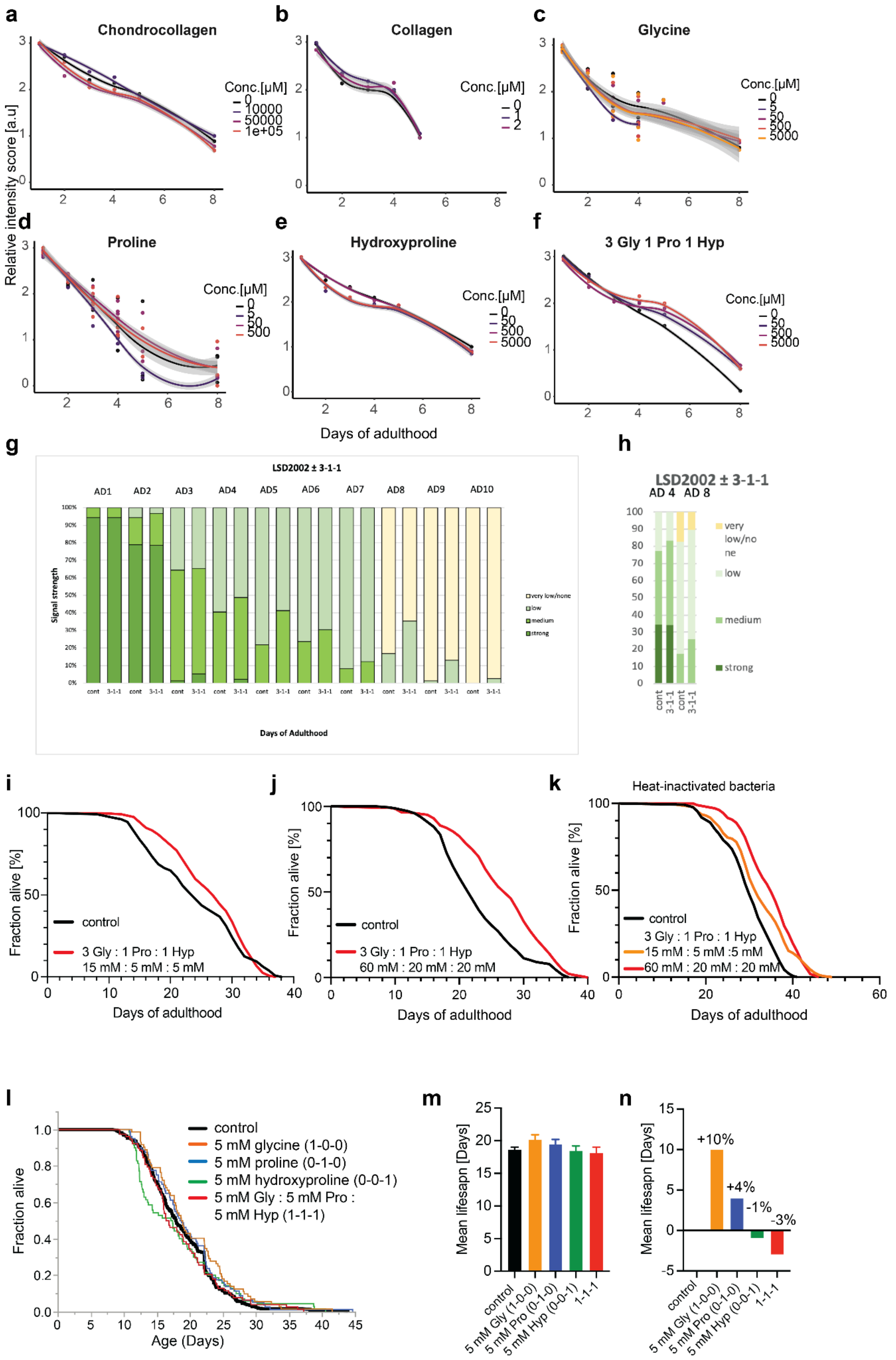
a
Chondrocollagen
Relative intensity score [a.u]
Conc.[μM]
0
10000
50000
1e+05
b
Collagen
Conc.[μM]
0
1
2
c
Glycine
Conc.[μM]
0
5
50
500
5000
d
Proline
Conc.[μM]
0
5
50
500
e
Hydroxyproline
Conc.[μM]
0
50
500
5000
f
3 Gly 1 Pro 1 Hyp
Conc.[μM]
0
50
500
5000
Days of adulthood
g
LSD2002 ± 3-1-1
AD1
AD2
AD3
AD4
AD5
AD6
AD7
AD8
AD9
AD10
Signal strength
very low/none
low
medium
strong
Days of Adulthood
h
LSD2002 ± 3-1-1
AD 4
AD 8
very low/none
low
medium
strong
cont
3-1-1
cont
3-1-1
i
Fraction alive [%]
control
3 Gly : 1 Pro : 1 Hyp
15 mM : 5 mM : 5 mM
Days of adulthood
j
Fraction alive [%]
control
3 Gly : 1 Pro : 1 Hyp
60 mM : 20 mM : 20 mM
Days of adulthood
k
Heat-inactivated bacteria
Fraction alive [%]
control
3 Gly : 1 Pro : 1 Hyp
15 mM : 5 mM :5 mM
60 mM : 20 mM : 20 mM
Days of adulthood
l
Fraction alive
control
5 mM glycine (1-0-0)
5 mM proline (0-1-0)
5 mM hydroxyproline (0-0-1)
5 mM Gly : 5 mM Pro :
5 mM Hyp (1-1-1)
Age (Days)
m
Mean lifesapn [Days]
control
5 mM Gly (1-0-0)
5 mM Pro (0-1-0)
5 mM Hyp (0-0-1)
1-1-1
n
Mean lifesapn [Days]
+10%
+4%
-1%
-3%
control
5 mM Gly (1-0-0)
5 mM Pro (0-1-0)
5 mM Hyp (0-0-1)
1-1-1

Supplementary Fig.1: Supplementation of 3 Gly : 1 Pro : 1 Hyp prolonged endogenous collagens expression during aging and promoted longevity.

a-f, Synchronized L4 transgenic *C. elegans* driving GFP with the collagen *col-144* promoter (LSD2002 P*col-144*::GFP) were placed on culturing plates containing heat-killed OP50 bacteria food with different concentrations of either chondrocollagen (**a**), rat tail collagen (**b**), glycine (**c**), proline (**d**), hydroxyproline (**e**), or a mixture of [3 Gly : 1 Pro : 1 Hyp] (F), and GFP intensity was scored during aging at 25°C. See Supplementary Table 1 for raw data. **g,h**, LSD2002 P*col-144*::GFP L4 were placed on heat-killed OP50 bacteria food with [3 Gly (15 mM : 1 Pro (5 mM) : 1 Hyp (5 mM)] (annotated as 311) and *col-144* expression was scored from 1-10 adulthood days (AD). For data and statistics, see Supplementary Table 1. **i-k**, Supplementation of [3 Gly : 1 Pro : 1 Hyp] starting from young adulthood increased the lifespan of temperature-sensitive wild-type background TJ1060 *C. elegans*. Concentration in mM given. TJ1060 *C. elegans* were placed on heat-inactivated bacteria treated with carbenicillin to exclude bacterial changes in the supplemented amino acids (**k**). **l-n**, Supplementation of individual amino acids or in a 1/1/1 ratio [1 Gly : 1 Pro : 1 Hyp] starting from young adulthood was insufficient to increase the lifespan of temperature-sensitive wild-type background TJ1060 *C. elegans*. **j**, Survival curves. **k**, Graph of mean +/- SEM lifespan. **l**, Graph of percent mean lifespan increase. For (**i-n**) statistical analysis, replicates and raw data are in Supplementary Table 2.

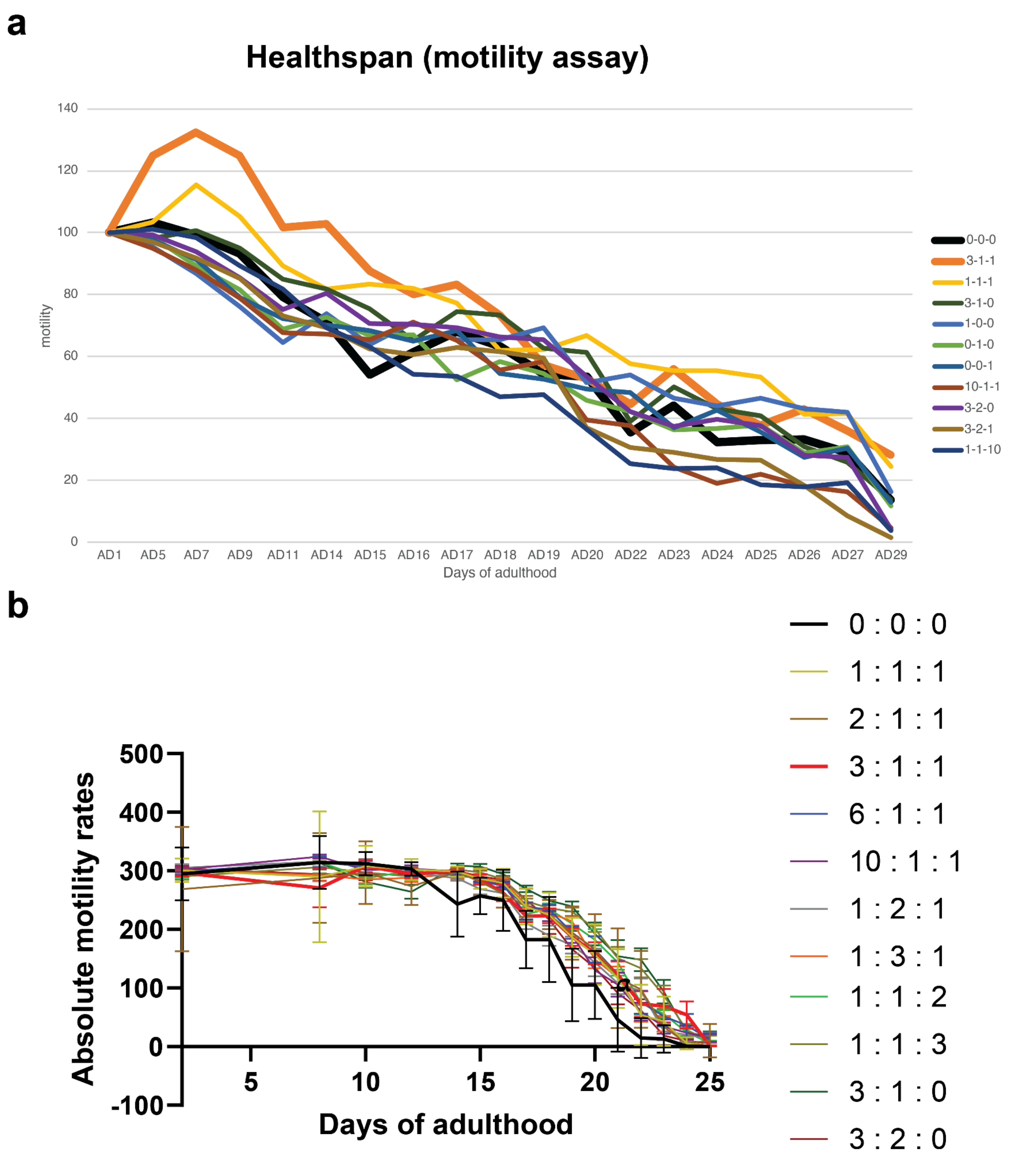

Supplementary Fig. 2: Longitudinal motility improved by 3 Gly : 1 Pro : 1 Hyp supplementation.

a-b, Synchronized day-1 adult *C. elegans* (**a**, TJ1060; **b**, N2) were placed into S-complete buffer containing heat-killed OP50 bacteria food with different ratios of [Gly : Pro : Hyp], and swimming speed (mobility) was measured as a proxy for healthspan. Raw data in Supplementary Table 3.

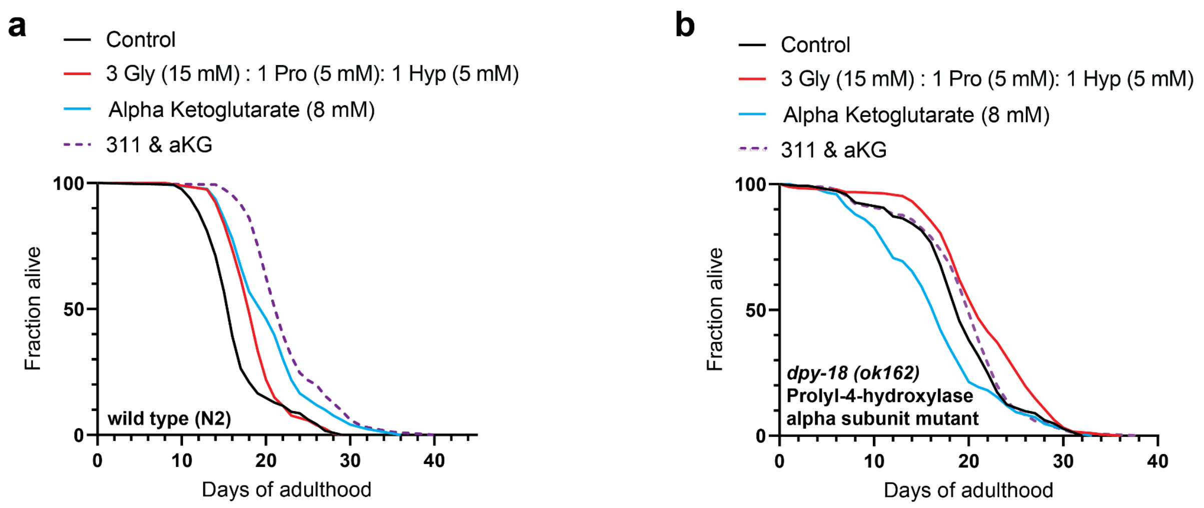

Supplementary Fig.3: The synergistic lifespan effects of 3 Gly: 1 Pro: 1 Hyp with alpha-ketoglutarate supplementation were mediated through two distinct mechanisms.

a, Supplementation of [3 Gly : 1 Pro : 1 Hyp], alpha ketoglutarate, or a combination starting from day 1 of adulthood increased the lifespan of wild type (N2) *C. elegans*.

b, Supplementation of [3 Gly : 1 Pro : 1 Hyp], but not alpha ketoglutarate, nor a combination starting from day 1 of adulthood, increased the lifespan of *dpy-18(ok162)* mutants. Lifespan data and statistics in Supplementary Table 2.

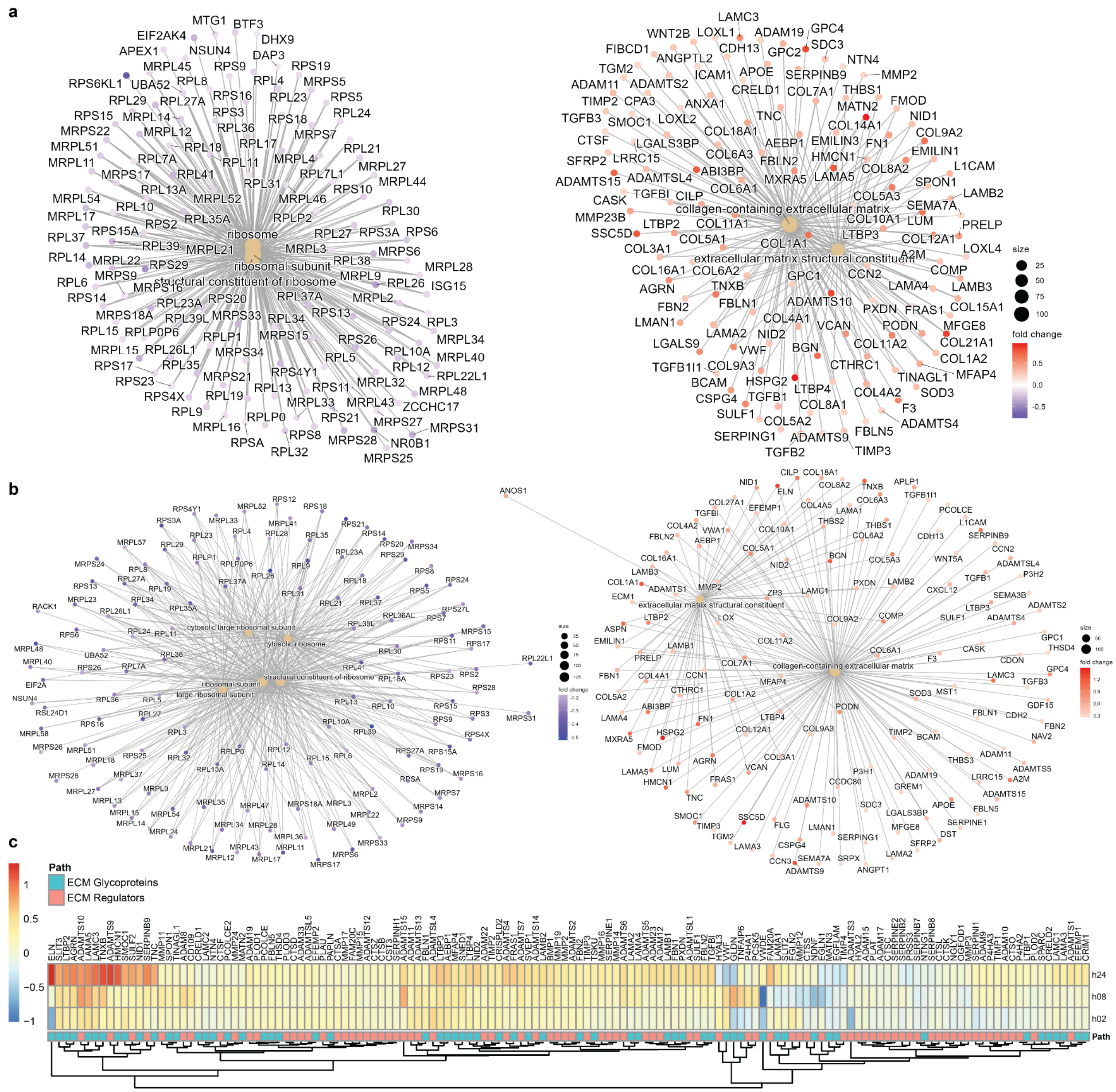

Supplementary Fig.4: Treatment of primary human dermal fibroblasts with 3 Gly : 1 Pro : 1 Hyp increased collagen-related gene expression.

a,b, Network plots of gene set enrichment analysis results after 8 hours (**a**) and 24 hours of treatment (**b**). **c**, Heatmap showing log2 fold change vs respective timepoint-matched controls of matrisome genes annotated as extracellular matrix (ECM) glycoproteins or ECM regulators.

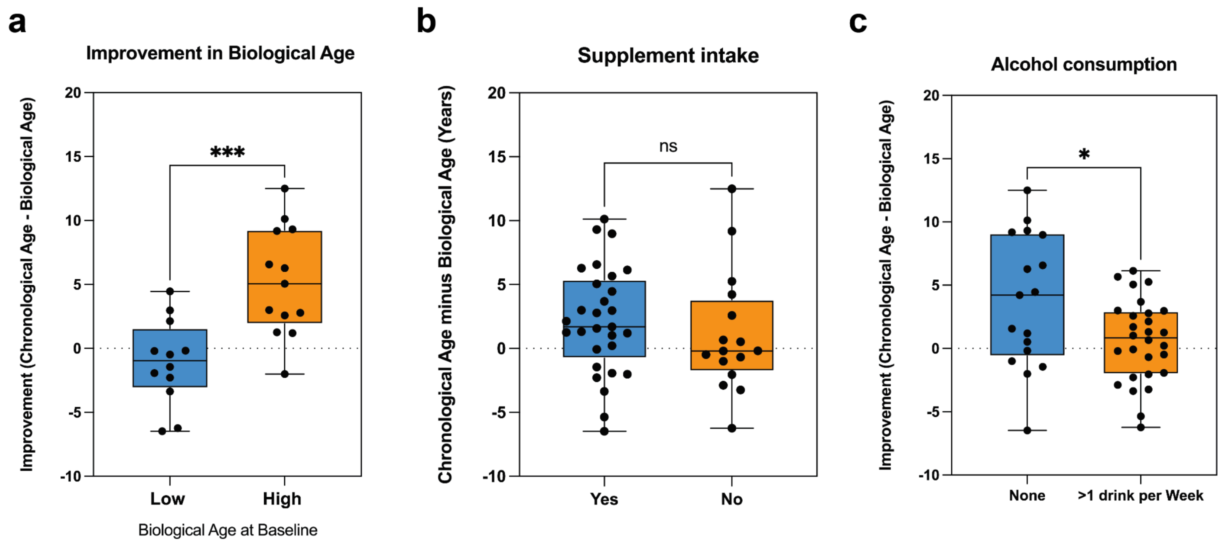

Supplementary Fig.5: Effects of initial biological age and lifestyle behavior on Collagen Activator's improvements on biological age.

a, Boxplot showing significant changes over 6 months in biological age for individuals with higher (25th percentile) and lower (75th percentile) biological age relative to their chronological age at baseline (P = 0.0004). **b**, Boxplot showing the distribution of biological age change over six months, stratified by supplement intake. No significant difference has been observed between the two groups. **c**, Boxplot showing the distribution of biological age change over six months, stratified by alcohol consumption. Alcohol drinkers showed less improvement in biological age than non-drinkers (P = 0.016).
